# Genomic and morphological data shed light on the complexities of shared ancestry between closely related duck species

**DOI:** 10.1038/s41598-022-14270-2

**Published:** 2022-06-17

**Authors:** Joshua I. Brown, Flor Hernández, Andrew Engilis, Blanca E. Hernández-Baños, Dan Collins, Philip Lavretsky

**Affiliations:** 1 Department of Biological Sciences, University of Texas at El Paso, El Paso, TX 79968 USA; 2 Museum of Wildlife and Fish Biology, Department of Wildlife, Fish, and Conservation Biology, University of California, Davis, One Shields Avenue, Davis, CA 95616 USA; 3 Department of Wildlife, Fish and Conservation Biology, University of California, Davis, One Shields Avenue, Davis, CA 95616 USA; 4 Departamento de Biología Evolutiva, Facultad de Ciencias, Universidad Nacional Autónoma de México, Mexico, Distrito Federal, Mexico; 5 U.S. Fish and Wildlife Service – Region 2 Migratory Bird Program, Albuquerque, NM USA

**Keywords:** Evolution, Population genetics, Speciation, Genetics, Animal physiology

## Abstract

Causes for genomic and morphological similarities among recently radiated species are often multifaceted and are further convoluted among species that readily interbreed. Here, we couple genomic and morphological trait comparisons to test the extent that ancestry and gene flow explain the retention of mallard-like traits within a sister species, the Mexican duck. First, we confirm that these taxa remain genetically structured, and that Mexican ducks exhibit an isolation-by-distance pattern. Despite the assumption of wide-spread hybridization, we found only a few late-stage hybrids, all from the southwestern USA. Next, assessing 23 morphological traits, we developed a genetically-vetted morphological key that is > 97% accurate in distinguishing across sex-age cohorts of Mexican ducks, mallards, and hybrids. During key development, we determined that 25% of genetically pure, immature male Mexican ducks of the northern population naturally displayed mallard-like traits in their formative plumage. In fact, applying this key to 55 museum specimens, we identified that only four of the 14 specimens originally classified as phenotypic hybrids were truly hybrids. We discuss how genomic and morphological comparisons shed light into the mechanism(s) underlying the evolution of complex phenotypic traits in recent radiations, and how misunderstanding the true morphological diversity within Mexican ducks resulted in taxonomic revisions that hindered conservation efforts.

## Introduction

Divergence and speciation proceed through four major evolutionary forces (i.e., mutation, selection, genetic drift, gene flow)^1–3^ that can be spatially and temporally variable and often result in genomic heterogeneity^4–6^. In recently diverged taxa where strong reproductive barriers have yet to develop, this kind of heterogeneity is common and can translate into phenotypic variation within and among populations^7,8^; this can make it challenging to discern the effects of hybridization vs. incomplete lineage sorting (ILS) throughout the genome^9–11^. Thus, resolving the true causes of phenotypic variability among incipient forms requires genomic and landscape-level sampling that allows us to accurately distinguish between phenotypic traits associated with hybridization versus shared ancestry^12^. Fortunately, advances in high-throughput sequencing methods have made it possible to conduct studies of closely related groups that require thousands of genetic markers to discern subtle population structure^11,13,14^.

Among lineages, class Aves generally shows high levels of shared ancestry due to its strong dispersal ability^15^, high chromosomal stasis^16^, and relatively slow development of post-zygotic breeding barriers^17,18^. These characteristics of avian evolution can result in species complexes that readily hybridize, as well as create discord in genomic and morphological divergence patterns^9–11,19^. Taxa within these groups often share large ancestral portions of their genome, which is readily passed between hybridizing groups, while only few genomic regions under directional selection are responsible for maintaining species boundaries^11,20–22^. For example, in the mallard complex, which consists of thirteen species of mallard-like ducks that have radiated around the world within the last 500,000 years^23^, taxa are morphologically diagnosable despite retaining large ancestral portions of their genomes^11,24^. Moreover, substantial proportions of these non-mallard (*Anas platyrhynchos*) taxa continue to display mallard-like phenotypic traits; however, distinguishing between introgression and ILS as a source for this phenotypic variability has been challenging (also see Lavretsky et al.^25^). Here, we use genetically vetted Mexican ducks (*Anas diazi*), mallards, and their hybrids to formally investigate the cause(s) of phenotypic variability in Mexican ducks, as well as identify phenotypic traits that are truly indicative of admixture.

### Study system

The Mexican duck is a non-migratory desert-adapted species of waterfowl endemic to the highlands of central Mexico extending north into the southwestern United States. While recently being re-elevated to full species^26^, the taxonomic status of the Mexican duck has been highly contentious because very little is known about their specific biology, ecology, and evolutionary history^27–30^. In particular, much of the debate surrounding their taxonomic status has been due to unknown rates of hybridization with mallards. Hubbard^27^ first described a clinal-like display of mallard-phenotypes (e.g., green in the head, curled tail-feathers, and a white neck ring) decreasing from north to south across the Mexican duck’s range resulting from high rates of hybridization with mallards. This became a major conservation concern, as persistent gene flow can lead to lineage fusion and genetic swamping^30,31^. However, more recent studies using double digest Restriction-site Associated DNA sequencing (ddRAD-seq) methods with hundreds of Mexican ducks and mallards revealed clear genetic structure between the two species and a general lack of genetic hybrids^11,20^. Furthermore, not only is there evidence of directional selection acting on a few regions throughout the genomes of mallards (e.g., Chromosomes 1, 2, and the Z-sex chromosome) and Mexican ducks (e.g., Chromosome 14)^11^, but that that the two species have been under divergent ecological selection^32^. Together, these findings established that these two species are under differing selective pressures, and that the presence of mallard-like traits in Mexican ducks might not be the result of hybridization. Given that many of the Mexican ducks with mallard traits were genetically identified as pure Mexican ducks, it seems likely that ILS is responsible for maintaining vestigial mallard characters in some Mexican duck populations^20^.

Here, we first build upon Lavretsky et al.^20^ to understand population structure within and between Mexican ducks and mallards by filling in geographical sampling gaps (i.e., Chihuahua, Sinaloa, and Southwestern USA). Although hybridization was thought to be most prevalent in the Mexican duck’s northern range, these estimates were based on phenotypic characters and may be overestimating true hybridization rates (i.e., ~ 2%; Lavretsky et al.^20^). In fact, given the decreasing number of mallards wintering in the southwestern US, and that mallards would have to drop out of migration (i.e., remain on their wintering grounds throughout the breeding season), for hybridization to occur, hybridization is likely rarer than previously thought^30,33^. Furthermore, we posit that hybridization is likely due to interbreeding with feral mallards, which have become a primary cause of interspecific hybridization throughout North America due to their increasing prevalence, aggressive breeding behaviors, and an extended breeding season^11,25,34–36^. We also aim to determine whether Mexican duck populations found in the Mexican states of Sonora and Sinaloa originated via founder events from their geographically closest interior Mexico population. Finally, for the first time, we use genetic vetting to describe morphological traits diagnostic of pure parentals and their hybrids. In doing so, we formally test whether the presence of mallard-like traits in Mexican ducks (e.g., green in the head, curled tail, black rump, etc.) is largely the result of hybridization or ancestry.

## Methods

### Sampling, DNA extraction, and ddRAD-seq library preparation

**Figure 1:**
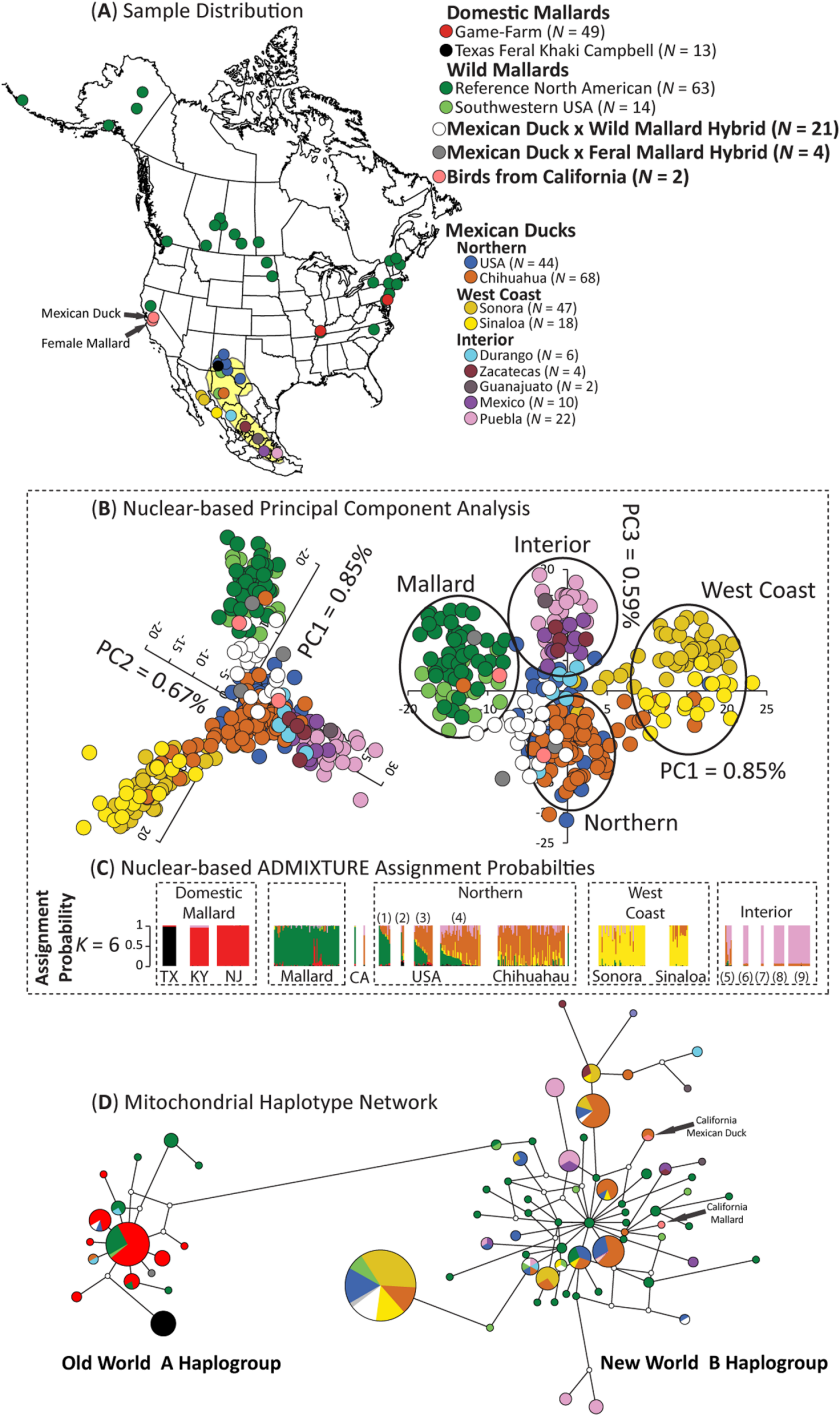
(**A**) Sample size and distributions of samples. (**B**) PCA and (**C**) ADMIXTURE assignments of sampled Mexican ducks, mallards, and putative hybrids, based on 12,696 bi-allelic ddRAD-seq nuclear SNPs. Numbered parentheses refer to different sample sites within the genetic cluster. Note that these analyses were based on 370 samples that excluded domestic mallards (Supplementary Materials Fig. S2) and all but one representative of each identified sibling group (Supplementary Materials Fig. S3). Finally, (**D**) a haplotype network based on 628 sequenced base-pairs of the mitochondrial control region. Note that we identify mallards by origin (wild vs. domestic) and Mexican ducks by geographical location in PCA and mitochondrial haplotype network, whereas assignment probabilities are colored by the six genetic clusters as estimated with ADMIXTURE.

We analyzed a total of 387 Mexican ducks, mallards, and their putative hybrids. In addition to previously published raw ddRAD sequences of Mexican ducks (N = 104; Lavretsky et al.^20^), we filled in geographical gaps by sampling tissue or blood from individuals collected in the Mexican states of Chihuahua (N = 68) and Sinaloa (N = 18), and in the southwestern USA (N = 59) (Fig. 1; https://github.com/jibrown17/MEDU_Metadata). In addition, a total of 138 wild (N = 76), domestic game-farm (N = 49), and feral Khaki Campbell (N = 13; i.e., a domestic breed known for their khaki-colored plumage) mallards collected throughout North America were also included in analyses, which included newly collected and previously published data^11,20,25,32^ (also see sample specifics in https://github.com/jibrown17/MEDU_Metadata). Note that game-farm mallards are domestic mallards being released on shooting preserves for hunting and/or dog training purposes and are now known to be the primary instigators of hybridization for wild populations of mallards and other mallard-like ducks^25^. Moreover, we used a group of feral Khaki Campbell mallards that were sampled alongside Mexican ducks and wild mallards as a genetic reference for other domestic mallards. Having wild and domestic mallards allowed us to determine which of these posed the highest risk for hybridization with Mexican ducks. Finally, two potential vagrant Mexican ducks collected in California were opportunistically sampled (Fig. 1A). Figures 1A, 4A and B have been derived by georeferencing and modifying publicly available USGS maps (https://www.sciencebase.gov/catalog/item/4fb555ebe4b04cb937751db9) using ArcGIS Desktop 10.6 software (https://support.esri.com/en/Products/Desktop/arcgisdesktop/arcmap/10-6-1).

This research was conducted under IACUC approval 967486-1 (University of Texas at El Paso) and USFWS scientific collecting permit MB11579C-3. All procedures involving animals were carried out in accordance with relevant guidelines and regulations.

Genomic DNA was extracted from blood or tissue using a DNeasy Blood & Tissue kit following the manufacturer’s protocols (Qiagen, Valencia, CA, USA) for the newly collected 32 wild mallards and 146 putative Mexican ducks (see sample-specific SRA data in https://github.com/jibrown17/MEDU_Metadata). DNA quality was visually assessed on a 1% agarose gel to ensure high molecular weight bands, and quantified using a Qubit 3 Flourometer (Invitrogen, Carlsbad, CA, USA) to ensure a minimum concentration of 20 ng/μL. ddRAD-seq library preparation followed protocols outlined in DaCosta and Sorenson^37^ (also see Lavretsky et al.^20^). In short, genomic DNA from individual samples was enzymatically fragmented using SbfI and EcoRI restriction enzymes. Next, Illumina TruSeq compatible barcodes were ligated to each sample for future de-multiplexing. Libraries were quantified, pooled in equimolar volumes, and the multiplexed library sent to the University of Oregon Core Genomics Facility for 150 base-pair, single-end chemistry sequencing on an Illumina HiSeq 4000 (detailed methods can be found in Supplementary Document S1).

Raw Illumina reads from previously and newly sequenced samples were de-multiplexed and processed using the computational pipeline described by DaCosta and Sorenson^37^ (Python scripts available at http://github.com/BU-RAD-seq/ddRAD-seq-Pipeline; also see Lavretsky et al.^20^). Loci were parsed into chromosomes by using BLASTN v.2^38^ and mapped to a Mallard reference genome (Accession no. SS263068950–SS263191362)^39,40^. Detailed bioinformatics methods can be found in Supplementary Document S1. We also note that large sections of the Z-sex chromosome was found to be under directional selection in mallards, and was shown to have distinct population structure due to its unique evolutionary history as compared to autosomal chromosomes among North American mallard-like taxa^11,20^.

### Mitochondrial DNA

For the same 174 newly collected samples, primers L78 and H774 were used to PCR amplify and sequence 655 base pairs of the mtDNA control region^41,42^ following Sanger Sequencing methods described in Lavretsky et al.^24^. Sanger sequencing occurred on an ABI 3730 (Applied Biosystems, Life Technologies, Carlsbad, California, USA) at the University of Texas at El Paso Border Biomedical Research Center Genomic Analysis Core Facility. The 170 (of 174) new sequences that passed quality control were deposited in GenBank (See Accession numbers at https://github.com/jibrown17/MEDU_Metadata). Previously published mtDNA control region sequences were combined, aligned, and edited with our new sequences using the MUSCLE v.3^43^ algorithm in Sequencher v. 4.8 (Gene Codes Corporation, Ann Arbor, MI, USA). Finally, haplotype relationships were visualized with a median-joining haplotype network using the program NETWORK v. 4.5.1.0^44^.

### Nuclear population structure and estimates of molecular diversity

Nuclear population structure was based on independent bi-allelic ddRAD-seq autosomal single nucleotide polymorphisms (SNPs) and without using a priori assignment of individuals to populations or species. Bi-allelic SNPs were extracted from a concatenated fasta file of ddRAD-seq autosomal loci using a custom python script in plink format (i.e., ped & map files)^45^. Following, PLINK v1.07^46^ was used to filter for singletons (i.e., minimum allele frequency (–maf 0.005)), any SNP missing ≥ 20% of data across samples (–geno 0.2), as well as any SNPs found to be in linkage disequilibrium (LD) (–indep-pairwise 2 1 0.5). One of the two SNPs was randomly excluded if an LD correlation factor (r2) > 0.5 was obtained.

First, a Principal Components Analysis (PCA) was done in the R package Adegenet^47^ with the ‘dudi.pca’ function. Next, we used programs ADMIXTURE v. 1.3^48,49^ and fineRADstructure^50^, with the latter being shown to better differentiate between patterns of shared ancestry (i.e., ILS) and recent hybridization in mallard complex taxa^11^. Specifically, assignment probabilities based on major allele frequencies such as in ADMIXTURE can be complicated by close genetic relationship, including for taxa that show patterns of isolation-by-distance as within Mexican ducks^20^. Rather, fineRADstructure co-ancestry matrices are calculated based on the rarest SNPs that contributes the most information, which not only allows it to account for linkage among individual ddRAD loci, but it is better suited for studies of incipient species with very little genetic differentiation^11,50^. For these reasons, we obtained both individual assignment and co-ancestry assignments across samples in order to better differentiate shared versus introgressed ancestry. Details about dataset filtering and settings used in ADMIXTURE and fineRADstructure analyses can be found in Supplementary Document S1.

Finally, composite pairwise estimates of relative divergence (ΦST) and nucleotide diversity (π) for mtDNA, as well as Autosomal and Z-chromosome ddRAD-seq loci were calculated across species as well as between Mexican duck sampling groups using the package PopGenome^51^ in the program R.

### Morphological assessment, assignment, and key development

A total of 302 birds with available structural and plumage trait data were examined, that included 194 genetically vetted mallards, Mexican ducks, and hybrids (see specifics in https://github.com/jibrown17/MEDU_Metadata). Due to a lack of genetically vetted mallards within our original molecular dataset and the apparent issues of feral game-farm mallard introgression among wild populations in eastern North America^25^, mallard morphological diversity was largely established with plumage information from an additional 108 mallards collected across their primary breeding range in the central prairies of Canada and the United States (i.e., the prairie pothole region). In addition to comparing samples from areas where the two species do not come into contact and thus may not interbreed, these mallards can still be considered to be of genetically pure North American wild ancestry (Fig. 1A). Plumage was assessed from both photos, frozen birds, and study skin specimens of the Mexican ducks, mallards, and putative hybrids housed at the UTEP Biodiversity collections (UTEPBC), Smithsonian National Museum of Natural History (SNMNH), American Museum of Natural History (AMNH) and the Museum of Wildlife and Fish Biology at University of California Davis (MWFB). Age and sex were assigned across samples using a molt cycle-based aging system of Pyle^52^. Specifically, a bird was determined to be immature if they were in pre-formative molt, formative plumage, first pre-alternate molt, or first alternate plumage, whereas adult birds were those in pre-basic molt, definitive basic plumage, pre-alternate molt, or definitive alternate plumage. Morphological characterization of pure Mexican duck, pure mallard, or hybrids were based on categorization of each samples genetic assignment attained from our ddRAD-seq autosomal population structure analyses.

Morphological assessments largely followed procedures in Scott and Reynolds^30^ and Bielefeld et al.^53^, and key development followed methods developed for the Hawaiian duck (*A. wyvilliana*, A. Engilis and J. Eadie, University of California-Davis, unpublished data) and mottled duck (*A. fulvigula*)^53^. Data was collected on an initial suite of six structural and 16 plumage traits, as well as mass (Table 1). Statistical support for each structural trait and mass in distinguishing across age-sex classes among Mexican ducks, mallards, and hybrids was attained by running an ANOVA (*p* < 0.001). For plumage, we first created categories within each of the 16 traits that would explain all possible phenotypes observed during visual assessment of photos and study skins across specimens (Table 1). Specifically, each trait was assigned an ordinal score representing whether it exhibited the Mexican duck (null = 0) or mallard (alternative = 1) form of the trait. Non-binary traits were given additional ordinal scores. For example, all adult male Mexican ducks exhibit straight central tail feathers, whereas the amount of tail-feather curl varied among male mallards. Thus, we surmised that a hybrid male would have at least some curl to its central tail feather. In this case, the tail curl trait was assigned 3 possible ordinal scores that represented pure Mexican duck (0 = no tail curl), mallard (2 = full tail curl), and intermediate (1 = partial curl) (Table 1). Note, that the initial categorization of each trait was based on the variability identified by assessing a subset of samples with photos from Lavretsky et al.^20^, study skins from various museums, and stock photos from books^23^. Without a priori knowledge of the phenotypic variability expressed in hybrids, many traits were given multiple ordinal scores to capture potential plasticity of traits that may arise from ancestry and/or hybridization (e.g., percent green in head; Table 1). Next, we visually assigned an ordinal score for all 16 plumage traits across samples, and then (1) examined general structure among samples by analyzing all possible plumage traits with a priori species information in a PCA using the “prcomp” command within the R base package (R Team 2020), and (2) determined which set of traits best explained the variance across age-sex cohorts of Mexican ducks, mallards, and hybrids using a linear discriminant analysis (LDA) as implemented in the R MASS package^54^. The summation across ordinal scores provided a final plumage score (PS) assigned to each sample. Finally, the categorization of sex-age cohorts of genetically pure Mexican duck, pure mallard, and hybrids established a range of PS values for each group; the strength of the assessed traits were determined based on the concordance between PS and genetic assignments across samples.

**Table 1:** Plumage (N = 16), structural + mass (N = 7) traits assessed between Mexican ducks (MEDU), mallards (MALL), and hybrids (HYB).

Plumage trait	Assignment criteria	Sex	Age
Primary covert pattern	Buff edged = MEDU (0); Plain/Solid = Hybrid/MALL (1)	M	I, A
		F	I, A
Lesser covert pattern	Buff edged = MEDU (0); Plain/Solid = MALL (1)	M	I, A
		F	I, A
Greater coverts pattern	Buffy or part white across coverts = MEDU (0); Complete white across coverts = Hybrid/MALL (1)	M	I, A
		F	I, A
Speculum color	Green = MEDU(0); No green = Hybrid/MALL (1)	M	I, A
		F	I, A
Percent green in head	No green = MEDU (0); 1–25% (1); 26–50% (2); > 50% = MALL (3)	M	I, A
Overall face and neck	Slightly patterned = MEDU (0); Continuously strong pattern = Hybrid/MALL (1)	M	I, A
		F	I, A
Black spots around bill	Absent = MEDU (0); Present = MALL (1)	M	I, A
Overall back feather pattern & color	Chevron patterned or buff/brown edges = MEDU (0); Solid or light patterned = MALL (1)	M	I, A
		F	I
Scapular pattern	Chevron patterned or buff/brown edges = MEDU (0); Solid or light patterned = MALL (1)	M	I, A
		F	I
Rump	Brown w/ buffy chenrons & buffy edges = MEDU (0); Black w/ rufous chevrons w/ rufous edges = Hybrid (1); Solid black = MALL (2)	M	I, A
		F	I
Outer two tail feathers (color of outer edges)	Buff edged = MEDU (0); White edged MALL (1)	M	I, A
		F	I, A
Central tail feather curl	Not raised = MEDU (0); Slightly raised (1); > Half curl = MALL (2)	M	I, A
Breast feather pattern & color	Strong internal pattern = MEDU (0); Slight internal pattern w/whitish edges = MALL (1)	M	I, A
		F	I, A
Overall breast & belly feather pattern	Uniform = MEDU (0); Breast & belly different color = MALL (1)	M	I, A
		F	I, A
Flank feather pattern	Chevron patterned = MEDU (0); Slight internal marking = MALL(1)	M	I, A
		F	I, A
Under-tail covert pattern	Chevron patterned = MEDU (0); Spotted to subtle internal marking = Hybrid (1); Gray—black or unpatterned = MALL (2)	M	I, A
		F	I, A

Structural Trait	Significantly different for		
Bill length	None significant		
Bill width	None significant		
Central tail feathers length	None significant		
Culmen-nares	None significant		
Tarsus length	Immature and adult female MEDU v. MALL; Adult male MEDU v. MALL		
Wing arc	Adult male MEDU v. MALL & Hybrid		
Mass	Adult male MEDU v. Hybrid v. MALL; Adult female MEDU v. Hybrid & MALL v. Hybrid		

All plumage traits found to be diagnostic are bolded, as well as the sex (Male = M; Female = F) and age (Immature = I; Adult = A) cohort for which the trait was diagnostic are identified. Assignment criteria, including species association are provided for each plumage. Finally, pair-wise comparison for which structural traits were significantly different (*p* < 0.01) are also provided—any unlisted comparisons are statistically insignificant.

Finally, we assessed 55 historical museum skins of putative Mexican ducks and hybrids deposited at the Museum of Southwestern Biology (MSB), and originally collected and classified by Huey^28^, and later by Hubbard^27^ to determine the biological and taxonomic status of the Mexican duck.

## Results

After filtering, we recovered a total of 3189 ddRAD-seq loci, with 3015 (270,895 (bp)) and 174 (15,609 bp) loci assigned to the autosomal and Z-sex chromosomes, respectively. Our dataset consisted of an average median sequencing depth of 128 (range = 26–687) reads per locus per sample, and an average of 97% of alleles present per locus. Finally, 625 bp of overlapping mtDNA control region was sequenced for a total of 376 samples (of 387; https://github.com/jibrown17/MEDU_Metadata).

### Mitochondrial haplotype structure

Haplotype tree reconstruction using the mtDNA control region revealed the two known Old World (OW) A and New World (NW) B haplogroups (Fig. 1D)^55–57^. Consistent with Lavretsky et al.^25^, all domestic lineage mallards (game-farm N = 49; Khaki Campbell N = 13) carried OW A haplotypes. Notably, three Mexican ducks (1%), 22 wild mallards (31%), 1 (of 3) Mexican duck x Feral mallard hybrids, and two (of 20) Mexican duck x wild mallard hybrids contained OW A haplotypes as well. Nevertheless, the majority of genetically vetted wild mallards (69%; 50/72) and 99% of Mexican ducks were found within the NW B haplogroup (Fig. 1D). Based on a simple tally, wild mallards had the most unique haplotypes (N = 43), followed by Mexican ducks from Southwestern USA, Chihuahua, and then those for more southern Mexico, whereas those from the western populations of Sonora and Sinaloa both carried only five unique haplotypes.

### Genetic diversity

Nucleotide diversity of Mexican ducks and wild mallards was similar across ddRAD-seq autosomal (πMALL = 0.0063, πMEDU = 0.0062) and Z-chromosome (πMALL = 0.0027, πMEDU = 0.0026; Supplementary Materials Fig. S1A) loci, as well as mtDNA (πMALL = 0.013, πMEDU = 0.0083), respectively. Both game-farm (πAut = 0.0051, πZ-chrom = 0.0025, πmtDNA = 0.0) and Khaki Campbell (πAut = 0.0035, πZ-chrom = 0.0013, πmtDNA = 0.0011) domestic mallard types had lower genetic diversity across loci as compared to their wild counterparts (Supplementary Materials Fig. S1B). Nucleotide diversity was similar across Mexican duck sampling groups for both autosomal and Z-chromosome markers (avg. πAut = 0.0060, avg. πZ-chrom = 0.0025), but varied substantially within mtDNA (range πmtDNA = 0.0041–0.016). Specifically, western-coast populations (i.e., Mexican ducks sampled in the Mexican states of Sonora (π = 0.0044) and Sinaloa (π = 0.0041)) showed decreased diversity in mtDNA, which is consistent with a recent founder event (also see Lavretsky et al.^20^).

### Population structure

Composite estimates of Φ_ST_ between Mexican ducks and wild mallards were 13-fold higher for ddRAD-seq Z-chromosome linked (Φ_ST_ = 0.071) than autosomal (Φ_ST_ = 0.0092; Supplementary Materials Fig. S1A) loci, and concordant with previous estimates^11,20^. Additionally, we report a two-fold higher relative differentiation estimate between Mexican ducks and game-farm mallards (Φ_ST_ Autosomal = 0.12, Φ_ST_ Z-Chromosome = 0.14) versus feral park mallards (Φ_ST_ Autosomal = 0.23, Φ_ST_ Z-Chromosome = 0.29). Finally, estimates of differentiation between Mexican duck sampling groups is relatively similar across ddRAD-seq Z-chromosome linked loci (range Φ_ST_ = 0–0.026), ddRAD-seq autosomal loci (range Φ_ST_ = 0.0017–0.048), and mtDNA (range Φ_ST_ = 0–0.57), with increased mtDNA differentiation (Φ_ST_ = 0.099–0.57) among western-coast populations (Supplementary Materials Fig. S1A).

Next, analyzing the complete 387 sample set, co-ancestry analyses identified 29 samples making up 12 sibling groups (see Supplementary Materials Fig. S2 & https://github.com/jibrown17/MEDU_Metadata). All putative sibling groups indeed were caught at the same time and location, were often flightless juvenile individuals, and all carried the same respective mtDNA haplotype. For example, three northern Mexican ducks that were all flightless juveniles sampled at the same time from the same location (Bosque del Apache, New Mexico) were identified as siblings as they had higher-than-average estimates of individual co-ancestry with one another (Supplementary Materials Fig. S2). Given that the inclusion of full siblings strongly biased PCA and ADMIXTURE analyses, all but one representative per sibling group was subsequently excluded in final population structure analyses. In the end, PCA, ADMIXTURE, and fineRADstructure analyses were based on a total of 12,696 (of 13,835) independent bi-allelic ddRAD-seq autosomal SNPs for 370 samples.

**Figure 2:**
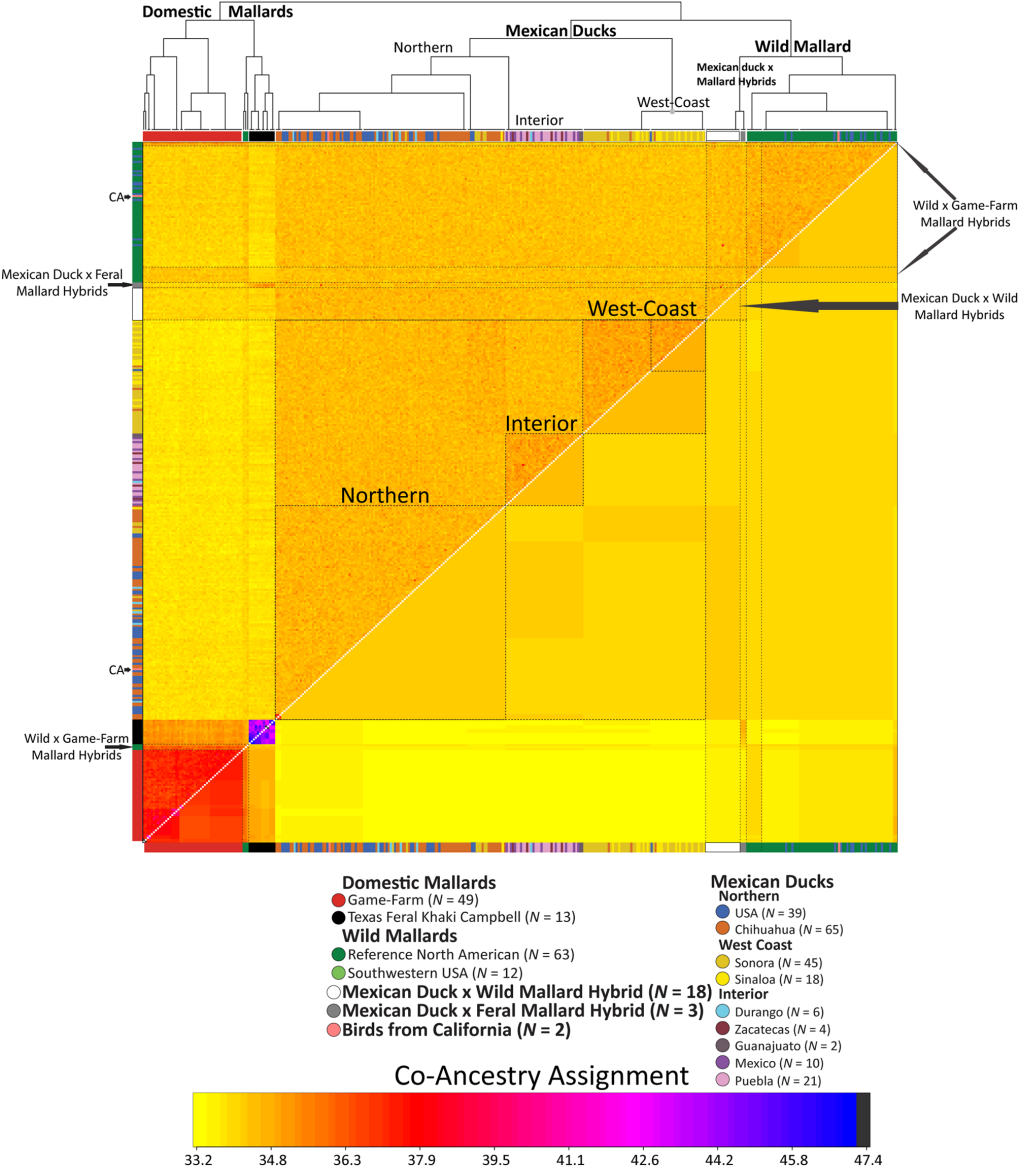
fineRADstructure individual (above diagonal) and average (below diagonal) coancestry coefficient matrix estimated using 12,696 bi-allelic ddRAD-seq autosomal SNPs. Note that this analysis was based on 370 samples that excluded all but one representative of each identified sibling group (Supplementary Materials Fig. S3).The level of recent coancestry is color coded from low (yellow) to high (blue). We color code mallards by origin (wild vs .domestic) and Mexican ducks by geographical location, as well as identify Mexican duck x (wild/feral) mallard hybrids and wild x game-farm mallard hybrids.

We visualized the first three principal components in our PCA, and individual assignment probabilities estimated in ADMIXTURE for K = 6 (Fig. 1B; Supplementary Materials Fig. S2). Despite recovering an optimum K of three, where two groups of domestic mallards were distinguished from Mexican ducks and wild mallards, additional structure was recovered by incrementally increasing the K to six (Supplementary Materials Fig. S2C). Given that we evaluated assignment probabilities at a population K value of six, bootstrap values and associated confidence intervals per sample were based on the sum assignment probability across mallard types (i.e., wild + game-farm + feral park), and the confidence intervals as the square root sum of each standard error squared^58^. Across all three analyses, we found wild mallards and Mexican ducks to be more closely related to one another than either were to domestic mallards, but that Mexican ducks and mallards remained genetically distinguishable from one another as well (Figs. 1 and 2; Supplementary Materials Figs. S2 and S3). In fact, < 1% of variation was explained by any single principal component (Fig. 1B; Supplementary Materials Fig. S2A–B). Within Mexican ducks, we recovered the three genetically unique populations: (1) a northern population comprised of the Mexican state of Chihuahua and Mexican ducks from the southwestern USA, (2) a western-coast cluster comprised of samples from the Mexican states of Sonora and Sinaloa, and (3) an interior Mexico population comprising samples from the Mexican states of Durango, Zacatecas, Guanajuato, Mexico, and Puebla (Fig. 1B). fineRADstructure showed a high degree of individual co-ancestry within these groups, as well as average co-ancestry to wild mallards that was on average higher than that recovered between domestic and wild mallards (Fig. 2; Supplementary Materials Fig. S3). Additionally, we find that western-coast Mexican ducks show a higher average co-ancestry with Mexican ducks from Chihuahua. Domestic mallards showed the highest levels of co-ancestry between individual samples, which is consistent with a history of domestication^25^. Finally, whereas one of the two putative Mexican ducks from California was simply a female mallard, the other was indeed a genetically pure Mexican duck that clustered with other Mexican ducks from Chihuahua based on both ADMIXTURE assignment probabilities and fineRADstructure co-ancestry estimates.

### Hybrid identification

First, ADMIXTURE identified a total of 53 samples that had substantial assignment (> 10%) to both Mexican ducks and the sum of all mallard types (i.e., domestic and wild). The majority of these were collected in southwestern USA (39/53, 74%), followed by Chihuahua (10/53, 19%), the western-coast regions of Sonora and Sinaloa (3/53, 6%), and a single sample from interior Mexico (1/53, 2%). In contrast, analyzing the full sample set with fineRADstructure identified a total of 25 samples, all of which were from the southwestern USA, with higher than average co-ancestry to both Mexican duck and mallard (wild or domestic) clusters. These samples were uniquely clustered between Mexican ducks and mallards and had a higher affinity to mallards (i.e., see dendrogram in Fig. 2; Supplementary Materials Fig. S3), suggesting these are indeed recent hybrids. Of the 25 samples, four had higher than average co-ancestry with feral Khaki Campbell mallards (Fig. 2). We note that an additional five samples were inferred to be hybrids through sibling relationships when analyzing the full dataset (Supplementary Materials Fig. S3).

Earlier caution on over-interpreting ADMIXTURE results (see above) is stressed as (1) mallards and Mexican ducks have a very close genetic relationship, (2) there is fine geographical-based structure within Mexican ducks, and (3) there have been recent founder events in western-coast states of Mexico (i.e., Sonora and Sinaloa). All of these factors can result in spurious assignments and forced us to analyze ADMIXTURE at a higher than optimum K (Supplementary Materials Fig. S1C). Additionally, we see the highest standard errors among Mexican ducks comprising the Northern genetic cluster, with the majority of them overlapping zero. Moreover, we found no association between an individual’s PS value and mallard assignment probability as determined by ADMIXTURE, which included individuals having ≤ 30% ADMIXTURE assignment to mallard, yet Mexican duck-equivalent PS values. Similarly, we found that individuals with > 50% ADMIXTURE assignment to mallard had mallard-equivalent PS values. Thus, in the case of the Mexican ducks, ADMIXTURE appears to be confounded by retained ancestry and is over-representing admixture (Fig. 1C; Supplementary Materials Fig. S2C). We conclude that fineRADstructure^50^ appears to be more reliable when dealing with recently diverged taxa that have high levels of shared ancestry, as well as with fine-scale geographical structure (e.g., isolation-by-distance among Mexican ducks; also see Lavretsky et al.^11^).

### Morphological assessment & assignment

For mass and the six structural traits evaluated here, we found at least one statistical difference among evaluated groups in only three traits (Supplementary Materials Fig. S4). First, tarsus length was statistically significant between Mallards and Mexican ducks when comparing immature females, as well as adult females and males. Mass was statistically different among adult male hybrids, mallards, and Mexican ducks, whereas adult female hybrids or Mexican ducks were statistically different from mallards. Finally, adult male Mexican ducks were statistically different from hybrids and mallards. Note that immature male Mexican ducks, mallards, and hybrids were not statistically different in any of the six structural traits. Such variance and increased missingness of structural traits caused errors in downstream analyses, and so structural traits were excluded in our PCA and LDA.

**Figure 3:**
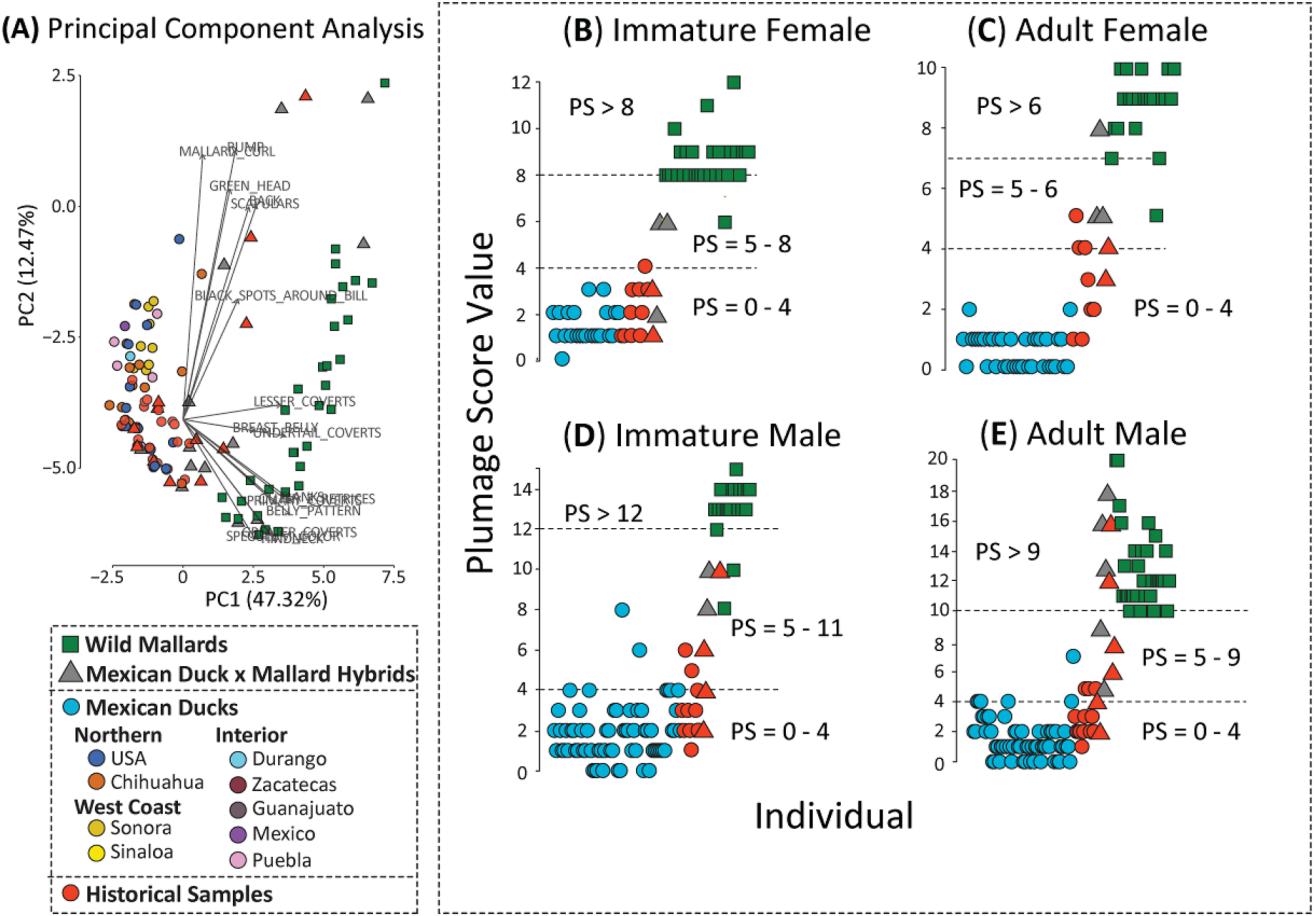
(**A**) PCA based on 16 plumage traits (Table 1) and assessed across 301 contemporary and 55 historical mallards, Mexican ducks, and hybrids. Scatter plots of Plumage Score (PS) values assigned across the same sample set, but specific to (**B**) immature females, (**C**) adult females, (**D**) immature males, and (**E**) adult males are provided. For each sex-age cohort, we demarcate specific PS ranges for Mexican ducks (0–4), hybrids (variable), and mallards (variable).

Our PCA showed clear separation between Mexican ducks and mallards based on the 16 assessed plumage traits (Fig. 3A; Supplementary Materials Fig. S5). Of the 25 genetic hybrids collected here, only 13 included morphological data; one and four clustered with Mexican ducks and mallards, respectively, while the remaining eight were recovered in intermediate space (Fig. 3B–E; also see sample specific scores in https://github.com/jibrown17/MEDU_Metadata). Next, LDA analyses recovered different optimum combinations of plumage traits as diagnostic for each age-sex cohort of Mexican ducks, mallards, and hybrids (Table 1; Supplementary Materials Fig. S6), and thus, a specific range of PS values for each comparison (Fig. 3B–E). In general, Mexican ducks were assigned PS values of ≤ 4 regardless of sex-age cohort (Fig. 3). Applying the developed keys (Supplementary Materials Document S2 & S3), we attained 100% and 97% accuracy in assigning across genetically-vetted sex-age cohorts of pure female and male Mexican ducks, respectively (Fig. 3). The four Mexican duck museum specimens that were identified with hybrid-equivalent PS values included two immature and one adult male Mexican duck (Fig. 3B–E). Importantly, however, only one adult female grouped with Mexican ducks, with the remaining 92% assigned as hybrid or mallard (Fig. 3B–E). The inability to distinguish hybrids and mallards may be due to the general lack of Mexican duck x mallard hybrids in our dataset^53^. Nevertheless, the developed key attained our ultimate goal to distinguish pure Mexican ducks from all others (Plumage Keys can be found in Supplementary Materials Document S2 & S3).

Finally, we applied respective sex-age cohort developed keys to the 55 historical specimens that originally included 41 and 14 putative Mexican ducks and Mexican duck x mallard hybrids^27^, respectively (Fig. 3). First, we find that 35 of the 41 historical samples originally characterized as Mexican ducks were correct (Fig. 3B–E). Of the six historical putative Mexican ducks with hybrid equivalent PS values (i.e., > 4), one was an adult female Mexican duck (Fig. 3C), two and three were immature (Fig. 3D) and adult (Fig. 3E) male Mexican ducks, respectively. Next, only 4 of the 14 (28.6%) original historical specimens identified as hybrids were considered as such when assessed with our keys. Of the remaining ten specimens, eight and two had PS values equivalent to pure Mexican duck or mallard, respectively (Fig. 3B–E). In the end, we find a total of ten historical samples that had hybrid equivalent PS scores, and determine that original assessments were highly inaccurate in hybrid identification.

## Discussion

**Figure 4:**
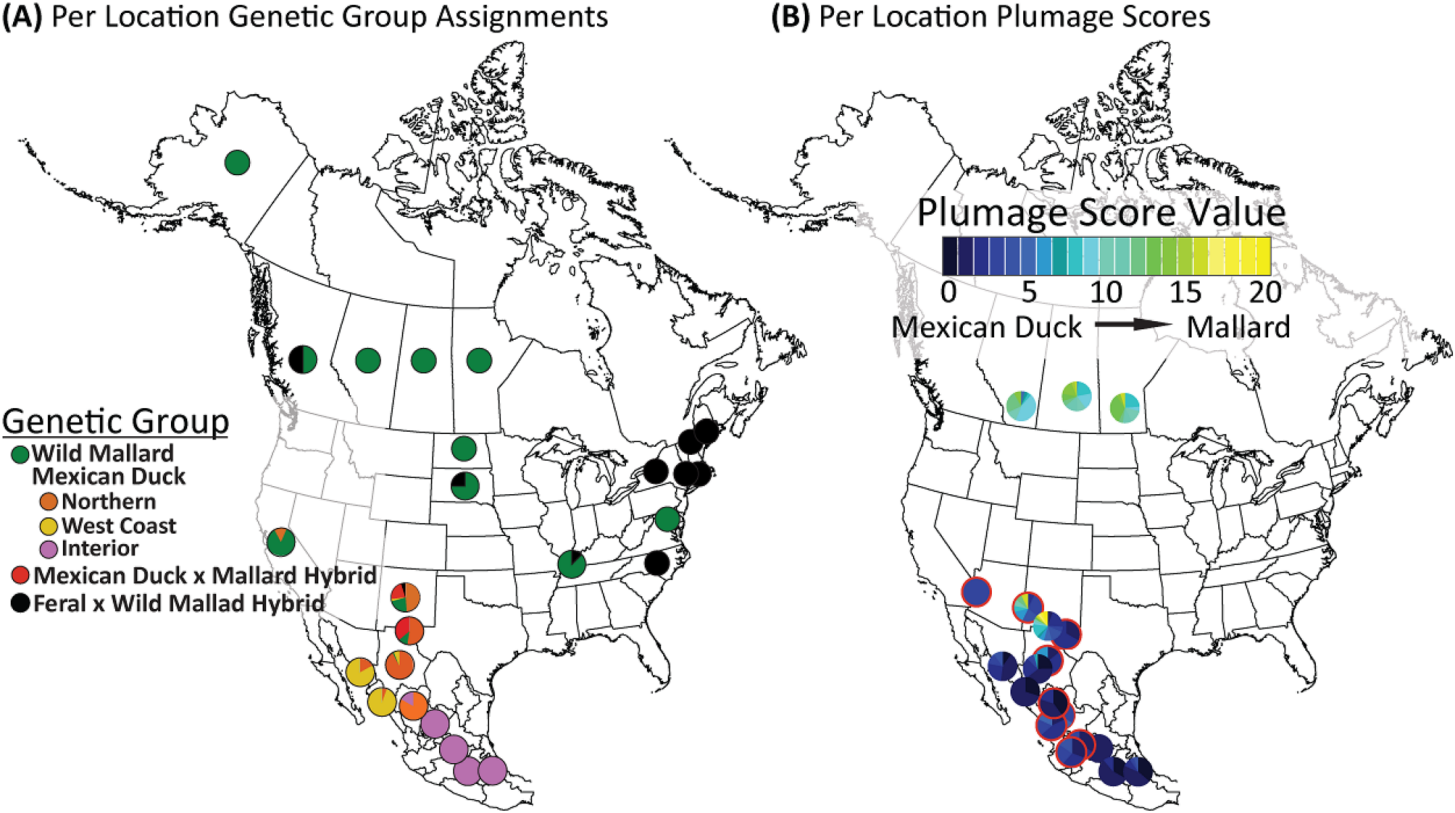
(**A**) Proportion of samples per geographical location with assignments to specific genetic groups as determined through population genetics analyses (Figs. 1 and 2; also see Supplementary Materials Figs. S2 & 3). (**B**) Plumage score proportions of samples per geographical location (Fig. 3; also see Supplementary Materials Fig. S6).

Increased genomic accessibility for non-model systems has allowed us to uncover evolutionary histories and subtle population structure within and between previously indistinguishable incipient forms^11^. Additionally, these methods permit for formal testing between potential causes of genetic and phenotypic trait retention among closely related taxa^59,60^. Whether ancestry, hybridization, or a combination of the two, cause retained genetic and morphological diversity among closely related species remains inferential unless genetically vetted. These questions are particularly complicated when only a portion of a population displays ancestral morphological traits, as the most parsimonious conclusion in such scenarios is to assume these are hybrids. Alternatively, such a scenario could result from stochastic retention of genetic variation and associated phenotypic traits, which have yet to be sorted among the incipient forms. For example, the clinal-like expression of mallard-like traits among Mexican ducks was hypothesized to be the result of extensive hybridization, to the point that Mexican ducks in the northern part of their range were thought to be a hybrid swarm^27,28,30,61^. However, genomic data presented here no longer support this conclusion. Rather, we provide strong evidence across the geographical range of the Mexican duck that hybrids are confined to southwestern USA, largely in human-dominated areas, and that formative males from this region naturally display a high proportion of mallard traits that appear to be subsequently lost as adults. Through our landscape level analysis we conclude that while Hubbard^27^ correctly identified the clinal-like variability in the display of mallard phenotypes across the Mexican duck’s range, he incorrectly concluded that it was due to high levels of hybridization with mallards. In fact, when applying our optimized plumage keys to historical samples from Hubbard^27^, we determined that 20% of immature males, 25% of adult males, and at least 57% of the hybrids were incorrectly identified (Fig. 3). Once re-evaluated, these historical samples show an almost identical distribution of PS values as samples collected here in the same respective regions (Fig. 4B). Thus, we conclude that the presence of mallard-derived genetic and phenotypic traits in Mexican ducks is the result of shared mallard ancestry and incomplete lineage sorting within Mexican ducks. Our study exemplifies the intricacies of species divergence, as well as emphasizes the importance of using genomic and morphological data jointly when attempting to disentangle ancestry and hybridization as a cause for trait (genetic and/or morphological) similarities among closely related taxa.

More generally, in concordance with evidence from Florida mottled ducks (*Anas fulvigula*)^53^, American black ducks (*Anas rubripes*)^62^, and Hawaiian ducks^63^, our results continue to support the hypothesis that the wild mallard was the ancestor to Mexican ducks and all North American monochromatic duck species^11,25,64^. While we note that Hawaiian ducks represent a unique case of hybrid speciation^35,63^, the evolution of mainland North American monochromatic species has likely resulted from the divergence of isolated pockets of mallards^25,32^. These kind of sequential divergence events of mallard-like species throughout North America, which have occurred over the 500,000 years, would explain the significant discord in genetic and phenotypic divergence found within these species^20,24,25^. Our study demonstrates the need for a holistic approach to truly understand such complex evolutionary histories as observed between Mexican ducks and wild mallards, as a single phenotypic or genetic marker type may yield spurious conclusions, which can have important implications for their conservation. For the Mexican duck, misunderstanding the cause for mallard-like phenotypes led to decades of taxonomic revisions and the belief that they were largely doomed due to introgressive hybridization^27,30,65^.

### Population structure of and shared phenotypic expression in Mexican ducks explained by retained wild mallard ancestry and not hybridization

In addition to providing the most up-to-date structural and plumage trait descriptions for the Mexican duck (Table 1), we used genetically vetted individuals to shed light on age-sex cohort variability of traits (Fig. 4B). Whereas adult male, adult female, and immature female Mexican ducks showed similar plumage patterns across their entire range, immature male Mexican ducks exhibited much greater variation. Specifically, we find that 25% (14 of 56) of genetically pure immature male Mexican ducks from the northern (~ 30%; 10 of 33) and western-coast (~ 17%; 4 of 23) genetic clusters innately express a higher proportion of mallard-like traits as compared to those from the interior Mexican ducks (i.e., 0%; Fig. 4B). In short, immature males can have all or a combination of ≤ 25% green in the head, unique tidal back patterns, black in the rump, vermiculation in the body, variable dark vermiculation in the under-tail feathers, and elevated tail curls (Table 1). Despite the problems that increased variability might cause in distinguishing hybrids, our plumage key for immature males resulted in 98% (179 of 182) assignment accuracy (Fig. 3D). In the end, we developed plumage keys for each age-sex cohort of Mexican ducks that can confidently (< 3% error rate) be applied to distinguish pure Mexican ducks from mallards/hybrids. While future work will require additional comparisons to determine whether modifications are needed, our efforts here resulted in the development of a plumage key that standardizes field identification of Mexican ducks (Supplementary Materials Documents S2 & S3), providing an important resource for citizen scientists and wildlife managers.

Although the evolution of dichromatism is often believed to be a derived state, an assessment of 977 species of birds suggests that there are many cases where dichromatism has been lost in favor of monochromatism^66^. However, in cases where dichromatism is lost, the underlying genetic diversity remains^66,67^, as dimorphic traits become controlled via modifiers (e.g., modifier alleles, steroids) rather than direct molecular changes^67–69^. In fact, the seasonal expression of dichromatic plumage in waterfowl are known to be estrogen-dependent^70^, where colorful plumage develops in the absence of testosterone/estrogen or luteinizing hormones^70–73^. Thus, monochromatic mallard-like ducks of North America that retain ancestrally derived mallard traits may control the expression of such plumage-linked genes through consistently elevated levels of testosterone or estrogen, which would induce monochromatic plumage year-round^74^. Regarding immature males, we hypothesize that unsuitable levels of testosterone prior to their pre-formative molt causes the partial expression of mallard-like traits in their first year. Similarly, older male and female monochromatic mallard-like ducks are known to regularly express male mallard-like plumage traits^67–69^, due to no longer being able to properly regulate hormone expression; this would explain the one adult male Mexican duck in our dataset that was identified as genetically pure, yet had a hybrid equivalent PS value (Fig. 3E). In general, New World mallard complex species provide a unique set of incipient forms that could act as a natural study system to understand the mechanisms underlying the evolution of complex phenotypic traits during the early stages of speciation as well as how expression of these traits is controlled during molt cycles.

### Low levels and confined Mexican duck x mallard hybridization

Although Mexican ducks have always been thought to be at risk of genetic swamping or extinction^27,30,65^, we contend that this has likely never been the case. We recovered few hybrids, all of which were confined to the northern part of the Mexican duck’s range (Figs. 2, 3 and 4). As predicted, we identified genetic hybrids primarily where Mexican ducks and mallards (wild and/or domestic) geographically co-occur during the breeding season. In fact, three of the four historical samples that possessed hybrid equivalent PS scores were found to be < 50 miles from large metropolitan areas. Thus, we caution that our results may in fact be overestimating hybridization rates due to the number of samples collected in more urban areas, where mallards (wild and feral) often remain year-round. While hybridization does occur, we conclude that it is relatively rare and largely confined to the southwestern USA (Fig. 4). Given that past taxonomic uncertainty was largely based on high rates of interbreeding with mallards, these results further support the recent taxonomic elevation of the Mexican duck to full species status^26^.

While we conclude that hybridization with mallards is currently not a major conservation concern, continued monitoring where locally breeding mallards (wild or domestic) occur is necessary. It will be particularly important to understand what proportion of hybridization is due to interactions with domestic mallards, as we provide evidence that at least four (of 25) putative hybrids were the result of pairings between Mexican ducks and feral park ducks (i.e., Khaki Campbell mallards). Additionally, we found three Mexican ducks carrying OW A haplotypes (Fig. 1D), which could only occur through a male Mexican duck and female domestic mallard pairing^25^. As domestic and not wild mallards continue to be found as the primary instigator of hybridization events among mallard complex taxa (Fig. 4A)^11,25,35^, educating the general public regarding the conservation consequences of domestic-lineage waterfowl releases is increasingly important.

### Mexican ducks evolved through sequential founder events

Landscape-level genetic sampling not only supports the three distinct Mexican duck genetic clusters previously reported by Lavretsky et al.^20^, but also fills in geographical gaps to expand our understanding of their population structure across the landscape (Figs. 1, 2 and 4A). In general, the fine-scale population structure recovered with nuclear DNA and mtDNA support recent findings that Mexican ducks evolved through sequential founder events^32^. Given that samples comprising the northern genetic cluster of Mexican ducks harbor all major mtDNA haplotypes (Fig. 1D) and are the combination of all nuclear variation found in other groups, we hypothesize that this is the ancestral population for all Mexican ducks, acting as the source for subsequent founder events into interior Mexico (Figs. 1C and 4A). In fact, this aligns with demographic and genotype-environment association models from Brown et al.^32^, which showed a cyclical pattern of population growth and contraction that generally corresponded with glacial and inter-glacial cycles, respectively^32^. Next, while we hypothesized that Mexican ducks from Sinaloa would be the natural extension of the closest interior populations, we actually find that both Sinaloa and Sonoran Mexican ducks are most similar to one another (Fig. 1D). Low mtDNA haplotype variation and strong partitioning in nuclear markers with other clusters is consistent with reports suggesting that Mexican ducks have only recently (i.e., ~ 1990) colonized Mexico’s western-coasts, likely from Chihuahua given the haplotype sharing between these groups (Figs. 1C, 2, and 4A)^20,75^. This westward expansion was likely in response to novel water availability due to the build-up of large dams, reservoirs, and canal systems for the rapid conversion of dry upland areas to intensive agriculture areas over the last 50 years in Mexico^76^.

Finally, we report for the first time a genetically pure Mexican duck collected in the Grasslands Area of Merced County, California; and while recently, an increasing number of Mexican ducks have been reported in California^77^, this is the furthest north this species has been genetically identified (Fig. 1). Surprisingly, while this bird was expected to be a vagrant from Mexico’s western-coast due to geographic proximity, this Mexican duck shared a mtDNA haplotype with and, based on nuclear loci, clustered with Chihuahuan samples (Figs. 1 and 2). Thus, this sample demonstrates that large-scale dispersal of Mexican ducks from their interior range is possible. While fine-scale population structure suggests that inter-population dispersal is infrequent (also see Lavretsky et al.^20^), the potential for and frequency of such migratory events to establish new populations outside of their current range remains unknown; in fact, it is possible that “vagrant” dispersal events may be how Mexican ducks expanded through time^78^. Finally, in addition to whole genome data, studies of Mexican duck ecology, short- and long-distance dispersal patterns, and life-cycle phenology are increasingly important for this recently elevated and unique North American waterfowl species.

## Supplementary Information

Supplementary Information 1.

Supplementary Information 2.

Supplementary Information 3.

Supplementary Information 4.

## Data Availability

GenBank accession numbers are publicly available at https://github.com/jibrown17/MEDU_Metadata. Other data files (e.g. FASTA files; IM, and STRUCTURE input files; Table of GenBank accession numbers): accession doi: 10.6084/m9.figshare.20032556.
