# Supplementary material for: Genomic and morphological data shed light on the complexities of shared ancestry between closely related duck species
Source: Sci Rep. 2022 Jun 17;12:10212. doi: 10.1038/s41598-022-14270-2 (PMC9205961; doi:10.1038/s41598-022-14270-2)
Supplement: Supplementary file 1 — Supplementary Information 1. [file 41598_2022_14270_MOESM1_ESM.docx]

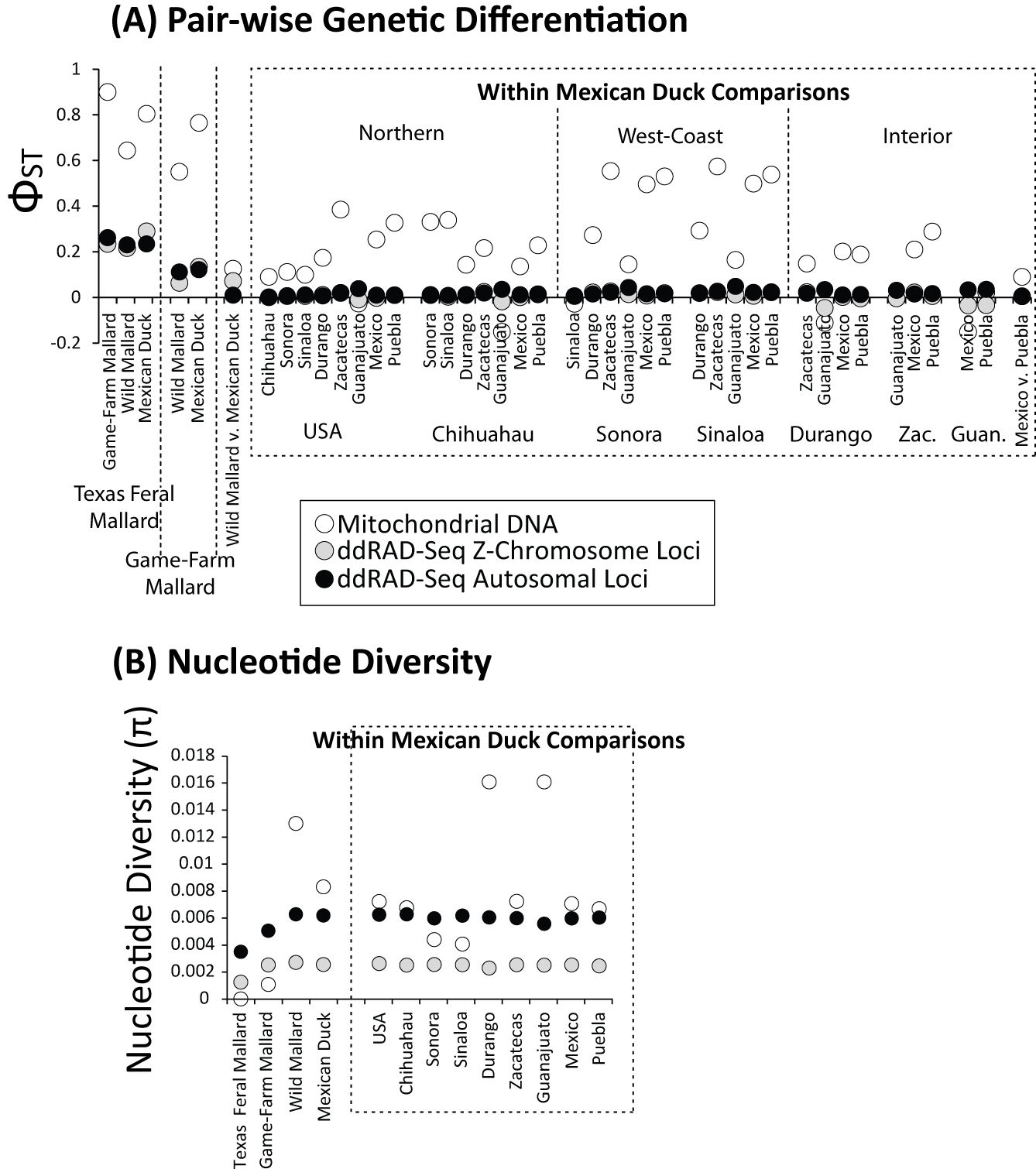
Figure S1. (A) Pairwise composite ΦST and (B) calculated nucleotide diversity estimated across 3,015 and 174 ddRAD-seq autosomal and Z-sex chromosome linked loci, respectively. Comparisons were done by species or domestic group, and within Mexican duck comparisons done by geographical location.


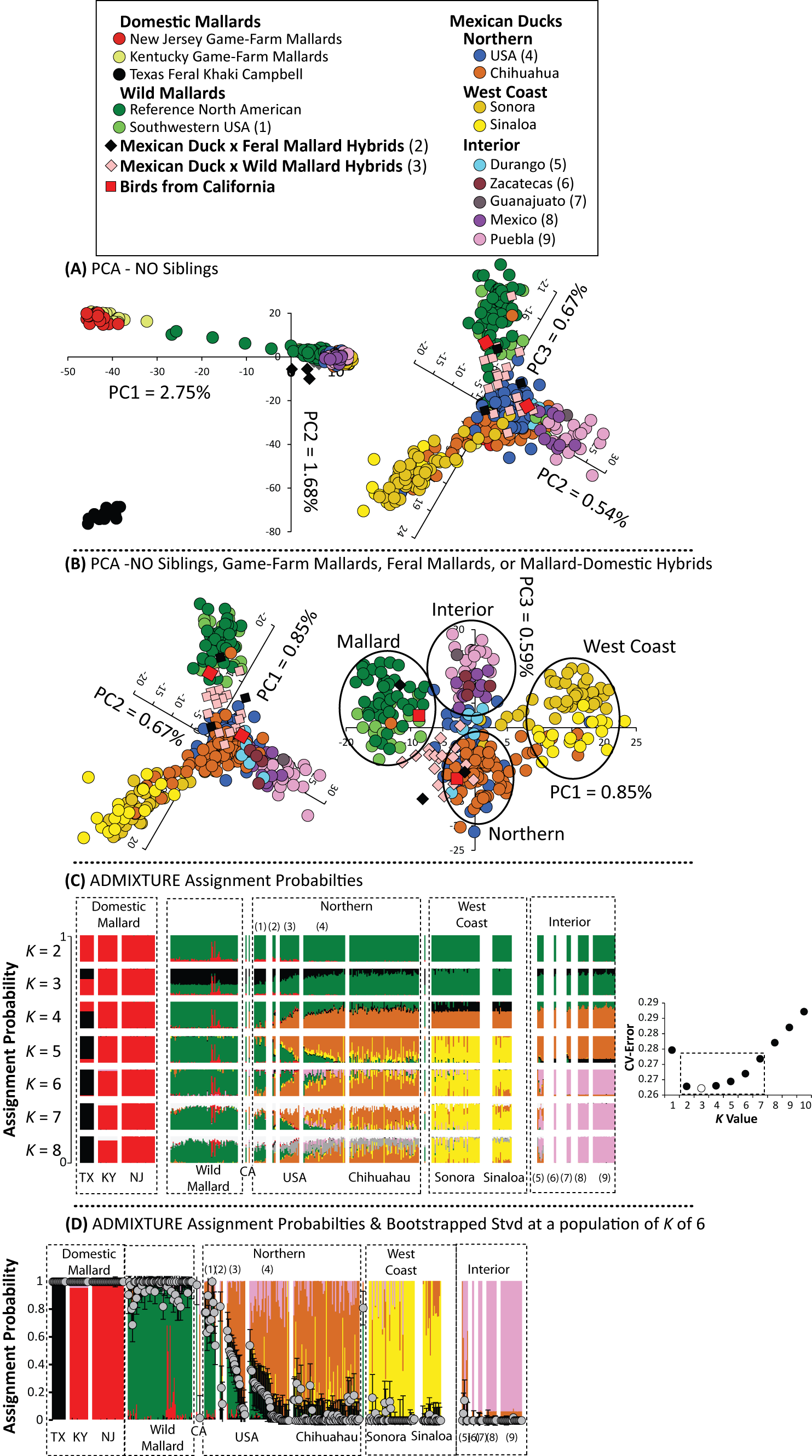


Figure S2. Population structure analyses of mallards (domestic and wild) and Mexican ducks, excluding all but one sample per identified sibling group (Supplementary Materials Figure S3), and using 12,696 independent bi-allelic ddRAD-seq autosomal SNPs. (A) PCA of all samples, identifying domestic mallards distantly clustering from wild mallards and Mexican ducks. (B) PCA excluding domestic mallards and siblings that identifies the four major clusters that Mexican ducks fall into. (C) ADMIXTURE assignment probabilities across samples for K populations of 2-8 and included respective CV-errors. (D) ADMIXTURE assignment probabilities analyzed under a K populations of 6 with overlapping bootstrapped average and standard deviation assignments to the summation of all possible mallard clusters (i.e., assignment probabilities to Feral Khaki Campbell , Game-Farm mallard, and wild mallard genetic clusters). Note that we identify mallards by origin (wild versus domestic) and Mexican ducks by geographical location in PCA, whereas assignment probabilities are colored by identified genetic clusters as estimated with ADMIXTURE for each population K value.


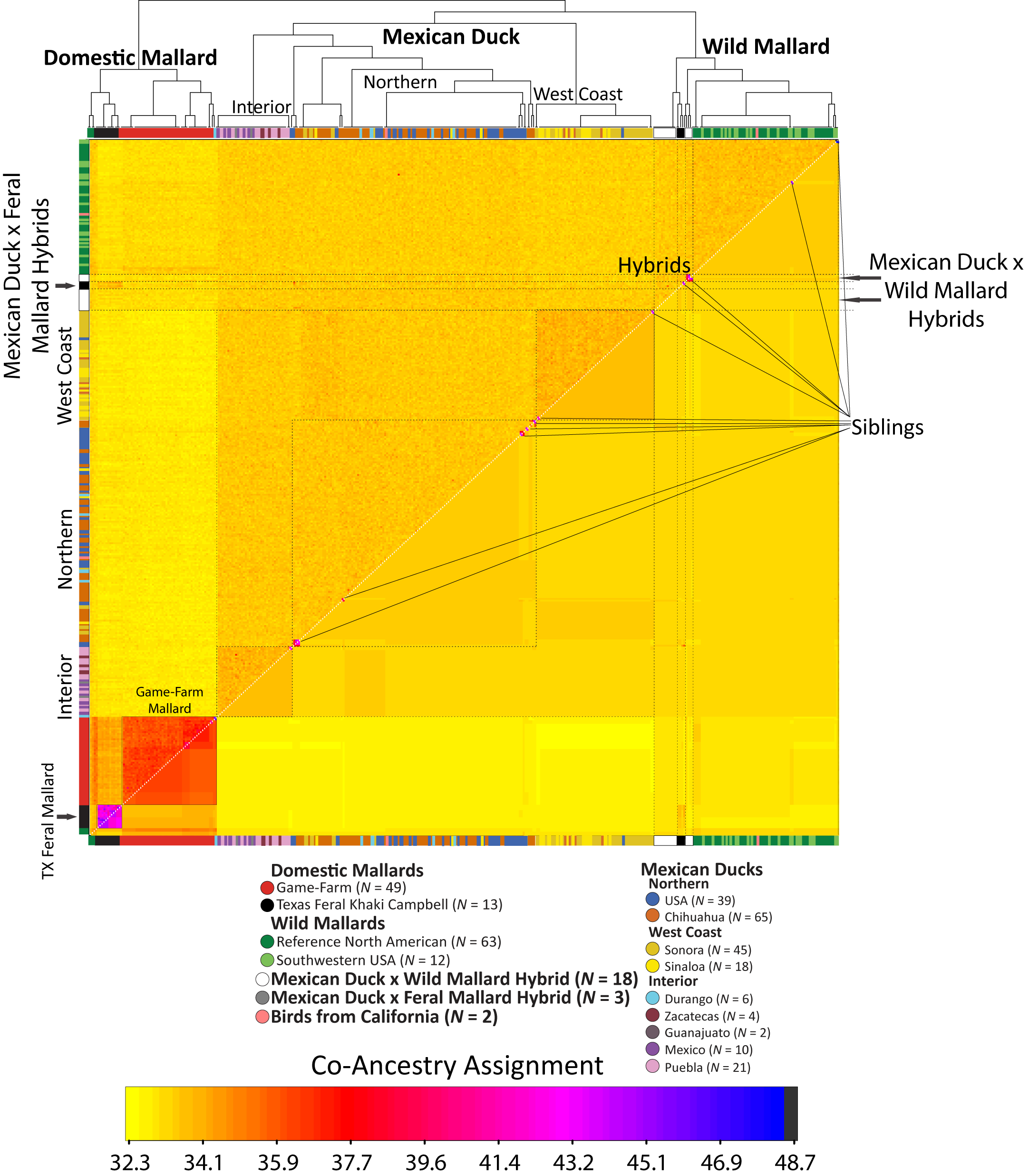
Figure S3. fineRADstructure individual coancestry coefficient matrix for the complete 387 sample dataset and based on 12,899 independent bi-allelic ddRAD-seq autosomal SNPs. The level of recent coancestry is color coded from low (yellow) to high (blue) is provided. We color code mallards by origin (wild versus domestic) and Mexican ducks by geographical location, as well as identify Mexican duck x (wild/feral) mallard hybrids, wild x game-farm mallard hybrids, and sibling groups.


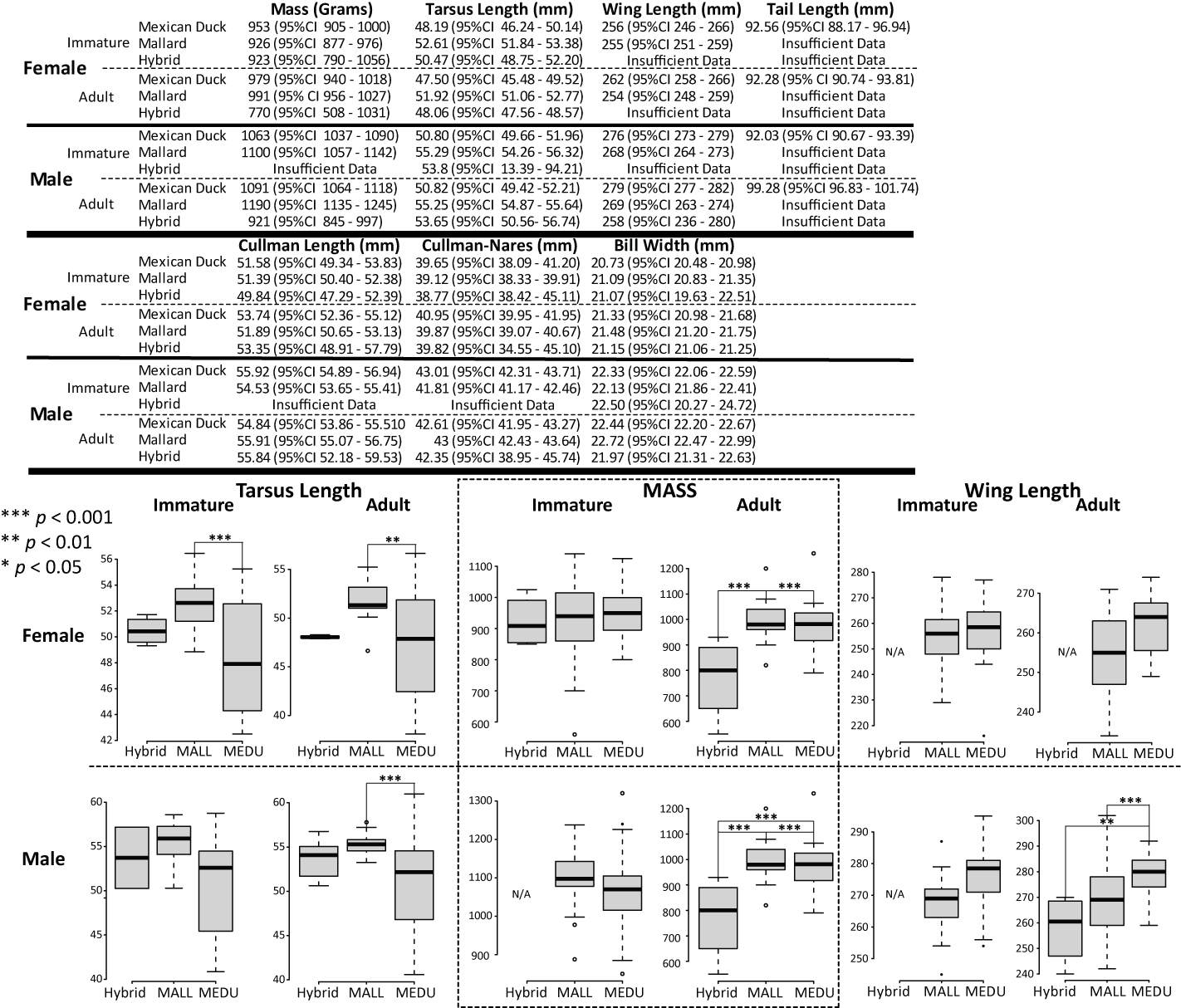
Figure S4. (Top) Average and 95% confidence intervals for mass and six structural traits assessed across Mexican ducks, mallards, and hybrids. (Bottom) Boxplot comparisons for the three traits with at least one statistically significant comparison.


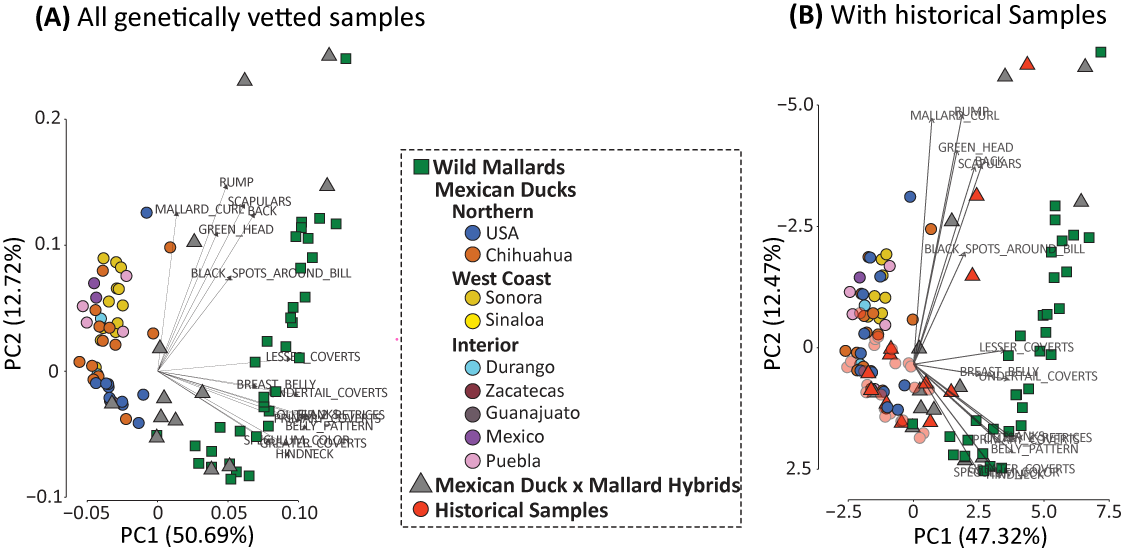
Figure S5. PCA analysis of all 16 plumage traits (Table 1) when analyzing (A) contemporary samples only, or (B) contemporary and historical samples. We identify wild mallards, hybrids, and historical samples, and with Mexican ducks identified by geographical location. Also, note that historical samples are color coded in red and either identified with original assignments as being Mexican duck (circle) or hybrid (triangle) for reference.


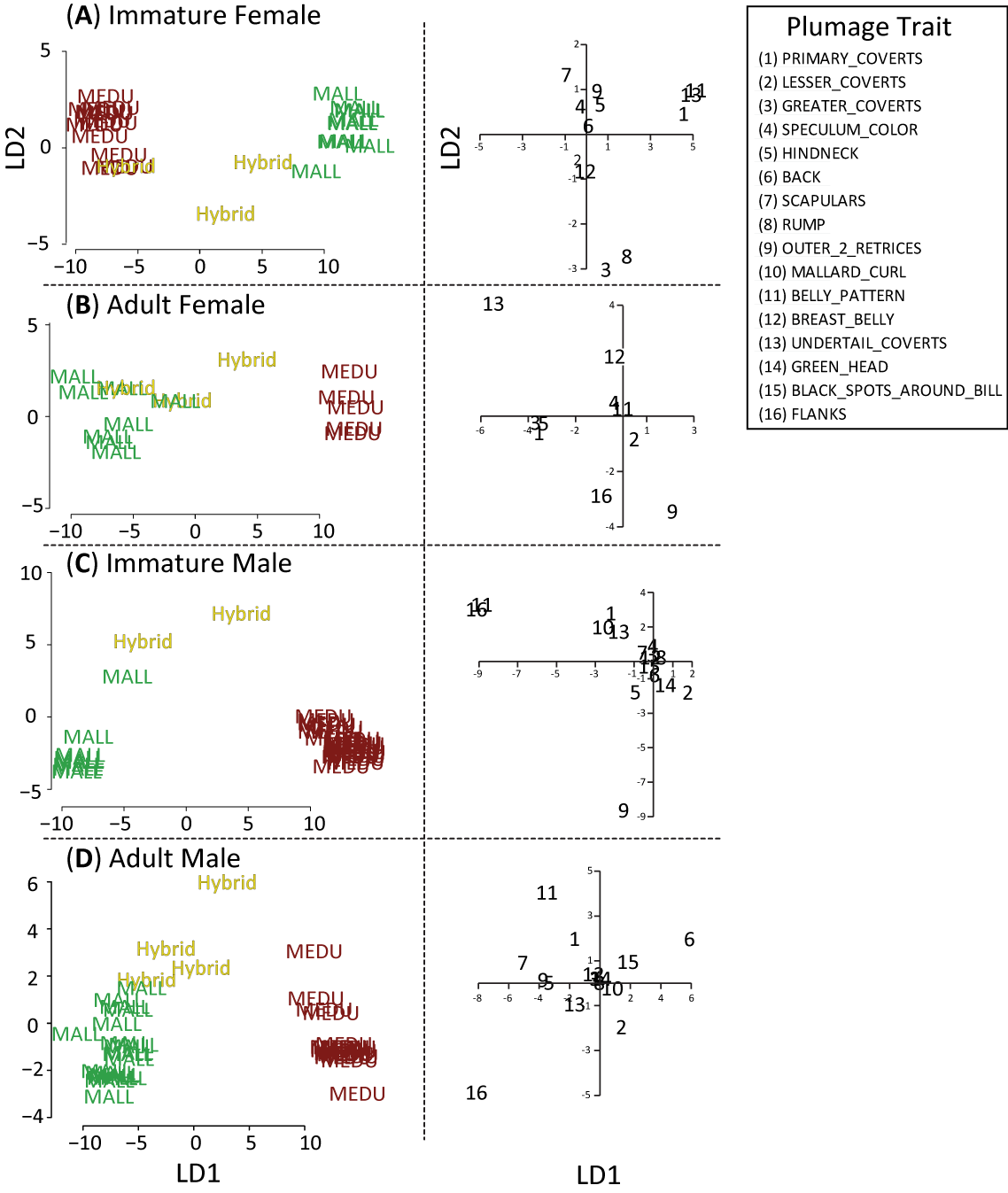
Figure S6. LDA results in capacity to assign between genetically vetted Mexican ducks, mallards, and hybrids and analyzed across sex-age cohorts. We include respective plumage trait PCAs, as these identify traits that provided the most information.
