# Supplementary material for: Genomic and morphological data shed light on the complexities of shared ancestry between closely related duck species
Source: Sci Rep. 2022 Jun 17;12:10212. doi: 10.1038/s41598-022-14270-2 (PMC9205961; doi:10.1038/s41598-022-14270-2)
Supplement: Supplementary file 2 — Supplementary Information 2. [file 41598_2022_14270_MOESM2_ESM.docx]

**Title: Genomic and morphological data shed light on the complexities of shared ancestry between closely related duck species**

Joshua I. Brown, Flor Hernández, Andrew Engilis, Jr., Blanca E. Hernández-Baños, Dan Collins, & Philip Lavretsky

**METHODS**

***Sampling, DNA Extraction, and ddRAD-seq Library Preparation***

We analyze a total of 387 Mexican ducks, mallards, and their putative hybrids. In addition to previously published raw ddRAD sequences of Mexican ducks (N = 104; Lavretsky et al., 2015), we filled in geographical gaps by including the Mexican states of Chihuahua (*N* = 67) and Sinaloa (*N* = 18), and increasing sampling effort in southwestern USA (*N* = 59) (Fig. 1; Supplementary Materials Table S1). Next, a total of 138 wild (*N* = 76), domestic game-farm (*N* = 49), and feral Khaki Campbell (*N* = 13) mallards were also included in analyses. Note that game-farm mallards are domestic mallards being released on shooting preserves for hunting and/or dog training purposes and are now known to be the primary instigators of hybridization for wild populations of mallards and other mallard-like ducks (P. Lavretsky et al., 2020). Moreover, we used the feral Khaki Campbell mallards as reference park mallards as were caught and sampled alongside Mexican ducks and wild mallards; and thus, had the potential to interbreed with wild ducks. Having wild and domestic mallards allowed us to determine which of these posed the highest risk for hybridization for Mexican ducks. Finally, two potential vagrant Mexican ducks collected in California were also opportunistically sampled (Figure 1A).

For a total of 174 new samples, genomic DNA was extracted from blood or tissue using a DNeasy Blood & Tissue kit following the manufacturer’s protocols (Qiagen, Valencia, CA, USA). DNA quality was visually assessed on a 1% agarose gel to ensure high molecular weight bands, and quantified using a Qubit 3 Flourometer (Invitrogen, Carlsbad, CA, USA) to ensure a minimum concentration of 20 ng/μL. ddRAD-seq library preparation followed protocols outlined in DaCosta and Sorenson (2014; also see Philip Lavretsky et al., 2015) (2014; also see Lavretsky et al. 2015a). In brief, genomic DNA was enzymatically fragmented using SbfI and EcoRI restriction enzymes. Illumina TruSeq compatible 6 base-pair barcodes were ligrated to allow for future de-multiplexing. The barcode-ligated fragments were then size selected for 300-450 bp fragments using gel electrophoresis (2% low-melt agarose), followed by gel purification using a MinElute gel extraction kit (Qiagen). Size selected fragments were then PCR amplified with Phusion high-fidelity DNA polymerase (Thermo Scientific, Pittsburgh, PA, USA) and purified with AMPure XP magnetic beads (Agencourt, Beverly, MA, USA). Libraries were quantified using a Qubit 3 Flourometer (Invitrogen, Carlsbad, CA, USA), pooled in equimolar, and the multiplexed library sent to the University of Oregon Core Genomics Facility for 150 base-pair, single-end chemistry sequencing on an Illumina HiSeq 4000.

Raw Illumina sequence reads were processed using the custom Python scripts designed by DaCosta & Sorenson (2014; Python scripts available at http://github.com/BU-RAD-seq/ddRAD-seq-Pipeline; also see Philip Lavretsky et al., 2015). Sequences that pass the preliminary Illumina quality filter were parsed into individual sample files based on barcode sequences before barcodes were trimmed from each read and replaced with a “CC” sequence to construct the SbfI recognition sequence. Low-quality reads were then filtered, and identical reads condensed while maintaining the read count and highest quality at each position. These condensed and filtered reads were then concatenated and clustered into loci using the UCLUST method in USEARCH v5 (Edgar, 2010) with an identity threshold of 0.85. Reads that had a quality score below 20 and do not cluster with other reads from the same individual at a 90% threshold were removed before further analysis. The highest quality read from each cluster was mapped to the Mallard reference genome (Accession no. SS263068950 – SS263191362; Huang et al., 2013; Kraus et al., 2011) using BLASTN v.2 (Altschul, Gish, Miller, Myers, & Lipman, 1990), and clusters with identical or nearly identical BLAST hits were combined while clusters that did not produce a BLAST hit were considered to be anonymous loci throughout the remainder of the pipeline. This step greatly improves the clustering of loci with large indel differences between samples. Then, MUSCLE v.3 (Edgar, 2010) was used to align sequences in each of the clusters (i.e. putative loci). Samples within each aligned cluster were genotyped using Python scripts written by DaCosta and Sorenson (2014). Alignments with end gaps due to indels and/ or a polymorphism in the SbfI restriction were trimmed or flagged for manual editing. Finally, using the Python script developed by Dacosta & Sorenson (2014), the aligned sequences were then genotyped. Homozygous genotypes were scored if >93% of reads were consistent with a single haplotype. Heterozygotes were scored if a second haplotype was represented by at least 29% of sequence reads. Samples with a secondary haplotype in 7-20% of reads, and putative heterozygote samples with a third haplotype in more than 10% of reads were flagged as ambiguous. Loci with multiple ambiguous genotypes are often representative of duplicated or repetitive elements in the genome. We then used the program Geneious (Biomaters Inc., San Francisco, CA, USA) to manually check flagged alignments and loci with unusually high numbers of polymorphisms or indels. To further limit the effect of sequencing error, we required a minimum sequencing depth of 5 reads to score an allele, such that a minimum of 10 reads was required to score a locus as homozygous or heterozygous. Loci with <15% missing genotypes were retained for downstream analyses, and final output files (e.g., FASTA, NEXUS, ADMIXTURE) were generated with custom python scripts (Philip Lavretsky et al., 2016). Moreover, ddRAD-seq loci with prefect BLAST scores to the reference genome were retained that allowed us to categorize loci as autosomal and Z-sex chromosome linked.

***Nuclear Population Structure and Estimates of Molecular Diversity***

Nuclear population structure was based on independent bi-allelic ddRAD-seq autosomal single nucleotide polymorphisms (SNPs) and without using a priori assignment of individuals to populations or species. Bi-allelic SNPs were extracted from a concatenated fasta file of ddRAD-seq autosomal loci using a custom python script in plink format (i.e., ped & map files) (Philip Lavretsky et al., 2016). Following, PLINK v1.07 (Purcell et al., 2007) was used to filter for singletons (i.e., minimum allele frequency (--maf 0.005)), any SNP missing ≥20% of data across samples (--geno 0.2), as well as any SNPs found to be in linkage disequilibrium (LD) (--indep-pairwise 2 1 0.5). One of the two SNPs was randomly excluded if an LD correlation factor (r2) > 0.5 was obtained.

First, a Principal Components Analysis (PCA) was done in the R package Adegenet (Jombart, 2008), with the ‘dudi.pca’ function. Next, we used programs ADMIXTURE v. 1.3 (Alexander & Lange, 2011; Alexander, Novembre, & Lange, 2009) and fineRADstructure (Malinsky, Trucchi, Lawson, & Falush, 2018), with the latter shown to better differentiate between patterns of shared ancestry (i.e., ILS) and recent hybridization in Mexican ducks (Philip Lavretsky, DaCosta, Sorenson, McCracken, & Peters, 2019). Specifically, assignment probabilities based on major allele frequencies such as in ADMIXTURE can be complicated by close genetic relationship, including for taxa that show patterns of isolation-by-distance as within Mexican ducks (Philip Lavretsky et al., 2015). Rather, fineRADstructure co-ancestry matrices are calculated based on the rarest SNPs that contribute the most information, which not only allows it to account for linkage among individual ddRAD loci but has been shown better suited for studies of incipient species that often have on average very little genetic differentiation as with Mexican ducks and Mallards (Philip Lavretsky et al., 2019; Malinsky et al., 2018). For these reasons, we obtained both individual assignment and co-ancestry assignments across samples in attempt to better capture shared versus introgressed ancestry.

Following the protocol outlined by Alexander et al. (2015; also see Philip Lavretsky et al., 2019), bi-allelic SNPs were extracted and formatted for ADMIXTURE analysis using the program Plink v. 0.67 (Purcell et al., 2007). For ADMIXTURE, we ran 100 iterations of each K for one through ten populations. The analysis uses a ten-fold cross-validation (CV) with a quasi-Newton algorithm (Zhou, Alexander, & Lange, 2011) and a block relaxation algorithm for point estimation. Each individual run was terminated once the change in log-likelihood (i.e., delta) of the point estimates increased by <0.0001. The optimal number of populations (K) was then based on the lowest averaged CV-error across all 100 replicates per K. The package PopHelper (Francis, 2017) in R was used to convert all ADMIXTURE outputs into CLUMPP v. 1.1 (Jakobsson & Rosenberg, 2007) input files. Final assignment probabilities were based on the optimal clustering alignment across all 100 replicates per evaluated population K value using the GreedySearch algorithm for 1000 iterations as implemented in CLUMPP v. 1.1. Additionally, confidence intervals (CI) were calculated for evaluated K population value through a 1,000 bootstraps (-B1000) as implemented in ADMIXTURE (Alexander et al., 2015).

Next, we used fineRADstructure (Malinsky et al., 2018) to more closely look at shared ancestry among Mexican ducks and mallards. Briefly, fineRADstructure identifies the most recent coalescent events among sample-by-sample pairwise comparisons to infer relatedness among individual samples and is informative in cases of recent and ongoing gene flow (Brown et al., 2020; Philip Lavretsky et al., 2019). Using the same set of bi-allelic SNPs for fineRADstructure, samples were assigned to populations using 1,000,000 iterations of the tree-building algorithm to assess genetic relationships among clusters. Results were visualized as heat maps using the provided R scripts fineradstructureplot.r and finestructurelibrary.r (available at <http://cichlid.gurdon.cam.ac.uk/fineRADstructure.html>).

**REFERENCES**

Alexander, D. H., & Lange, K. (2011). Enhancements to the ADMIXTURE algorithm for individual ancestry estimation. *BMC Bioinformatics*, *12*(1), 246. doi:10.1186/1471-2105-12-246

Alexander, D. H., Novembre, J., & Lange, K. (2009). Fast model-based estimation of ancestry in unrelated individuals. *Genome Research*, *19*(9), 1655–1664. doi:10.1101/gr.094052.109

Alexander, D. H., Shringarpure, S. S., Novembre, J., & Lange, K. (2015). *Admixture 1.3 Software Manual*. Los Angeles.

Altschul, S. F., Gish, W., Miller, W., Myers, E. W., & Lipman, D. J. (1990). Basic local alignment search tool. *Journal of Molecular Biology*, *215*(3), 403–410. doi:10.1016/S0022-2836(05)80360-2

Brown, J. I., Lavretsky, P., Wilson, R. E., Haughey, C. L., Boyd, W. S., Esler, D., … Sonsthagen, S. A. (2020). High site fidelity does not equate to population genetic structure for common goldeneye and Barrow’s goldeneye in North America. *Journal of Avian Biology*, *51*(12). doi:10.1111/jav.02600

DaCosta, J. M., & Sorenson, M. D. (2014). Amplification biases and consistent recovery of loci in a double-digest RAD-seq protocol. *PLoS ONE*, *9*(9), e106713. doi:10.1371/journal.pone.0106713

Edgar, R. C. (2010). Search and clustering orders of magnitude faster than BLAST. *Bioinformatics*, *26*(19), 2460–2461. doi:10.1093/bioinformatics/btq461

Francis, R. M. (2017). POPHELPER: an R package and web app to analyse and visualize population structure. *Molecular Ecology Resources*, *17*(1), 27–32. doi:10.1111/1755-0998.12509

Huang, Y., Li, Y., Burt, D. W., Chen, H., Zhang, Y., Qian, W., … Li, N. (2013). The duck genome and transcriptome provide insight into an avian influenza virus reservoir species. *Nature Genetics*, *45*(7), 776–783. doi:10.1038/ng.2657

Jakobsson, M., & Rosenberg, N. A. (2007). CLUMPP: a cluster matching and permutation program for dealing with label switching and multimodality in analysis of population structure. *Bioinformatics*, *23*(14), 1801–1806. doi:10.1093/bioinformatics/btm233

Jombart, T. (2008). adegenet: a R package for the multivariate analysis of genetic markers. *Bioinformatics*, *24*(11), 1403–1405. doi:10.1093/bioinformatics/btn129

Kraus, R. H., Kerstens, H. H., Van Hooft, P., Crooijmans, R. P., Van Der Poel, J. J., Elmberg, J., … Groenen, M. A. (2011). Genome wide SNP discovery, analysis and evaluation in mallard (Anas platyrhynchos). *BMC Genomics*, *12*(1), 150. doi:10.1186/1471-2164-12-150

Lavretsky, P., McInerney, N. R., Mohl, J. E., Brown, J. I., James, H. F., McCracken, K. G., & Fleischer, R. C. (2020). Assessing changes in genomic divergence following a century of human-mediated secondary contact among wild and captive-bred ducks. *Molecular Ecology*, *29*(3), 578–595. doi:10.1111/mec.15343

Lavretsky, Philip, Dacosta, J. M., Hernández-Baños, B. E., Engilis, A., Sorenson, M. D., & Peters, J. L. (2015). Speciation genomics and a role for the Z chromosome in the early stages of divergence between Mexican ducks and mallards. *Molecular Ecology*, *24*(21), 5364–5378. doi:10.1111/mec.13402

Lavretsky, Philip, DaCosta, J. M., Sorenson, M. D., McCracken, K. G., & Peters, J. L. (2019). ddRAD‐seq data reveal significant genome‐wide population structure and divergent genomic regions that distinguish the mallard and close relatives in North America. *Molecular Ecology*, *28*(10), 2594–2609. doi:10.1111/mec.15091

Lavretsky, Philip, Peters, J. L., Winker, K., Bahn, V., Kulikova, I., Zhuravlev, Y. N., … McCracken, K. G. (2016). Becoming pure: Identifying generational classes of admixed individuals within lesser and greater scaup populations. *Molecular Ecology*, *25*(3), 661–674. doi:10.1111/mec.13487

Malinsky, M., Trucchi, E., Lawson, D. J., & Falush, D. (2018). RADpainter and fineRADstructure: Population Inference from RADseq Data. *Molecular Biology and Evolution*, *35*(5), 1284–1290. doi:10.1093/molbev/msy023

Purcell, S., Neale, B., Todd-Brown, K., Thomas, L., Ferreira, M. A. R., Bender, D., … Sham, P. C. (2007). PLINK: A tool set for whole-genome association and population-based linkage analyses. *American Journal of Human Genetics*, *81*(3), 559–575. doi:10.1086/519795

Zhou, H., Alexander, D., & Lange, K. (2011). A quasi-Newton acceleration for high-dimensional optimization algorithms. *Statistics and Computing*, *21*(2), 261–273. doi:10.1007/s11222-009-9166-3
