# Supplementary material for: Genomic and morphological data shed light on the complexities of shared ancestry between closely related duck species
Source: Sci Rep. 2022 Jun 17;12:10212. doi: 10.1038/s41598-022-14270-2 (PMC9205961; doi:10.1038/s41598-022-14270-2)
Supplement: Supplementary file 3 — Supplementary Information 3. [file 41598_2022_14270_MOESM3_ESM.pdf]

Mexican Duck & Mallard Plumage Traits Scoring Key Version 2

Flor B Hernandez Camacho,
Andy Engilis Jr., & Philip Lavretsky

Basic Topography

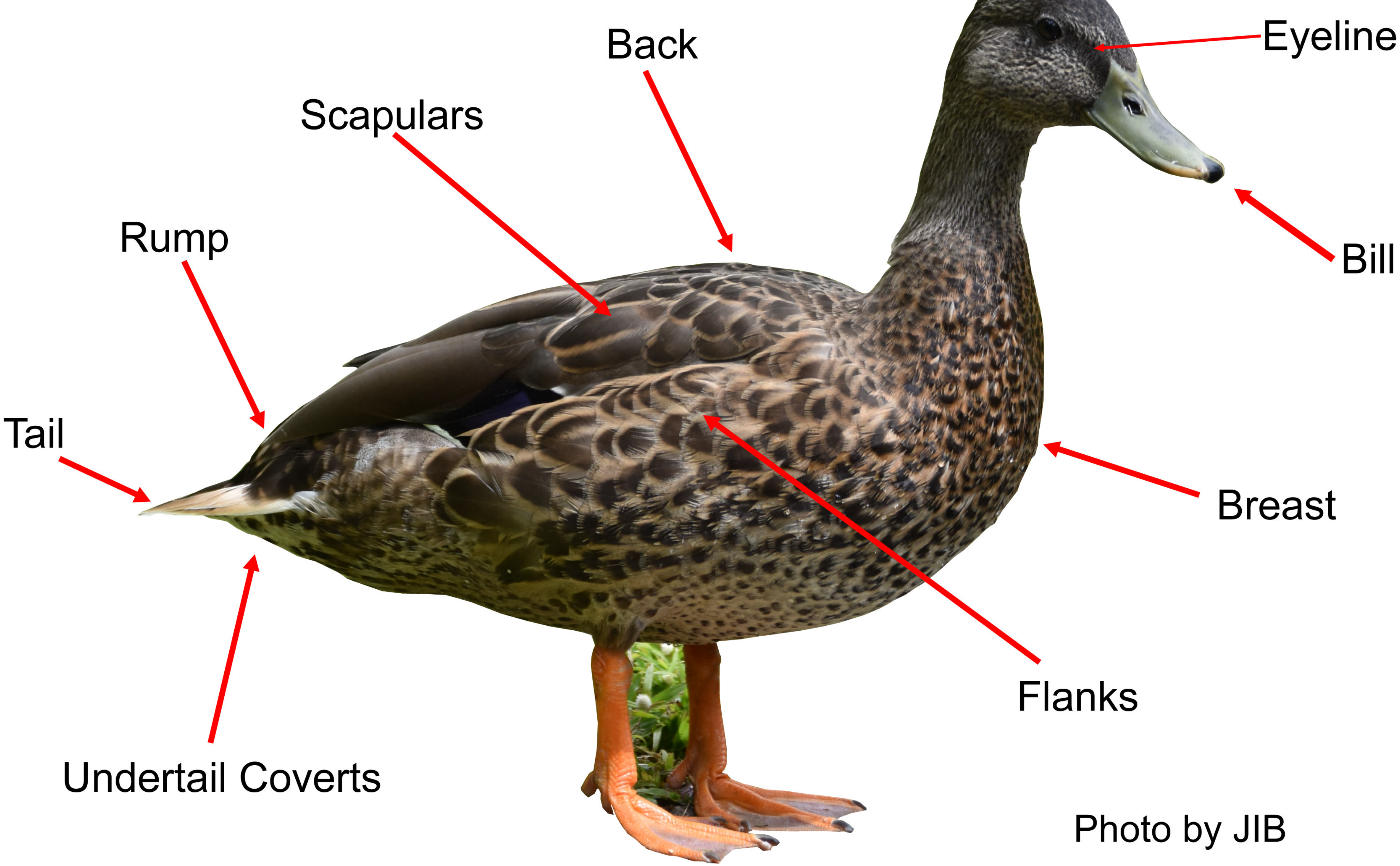

Photo by JIB

Basis topography of a duck wing

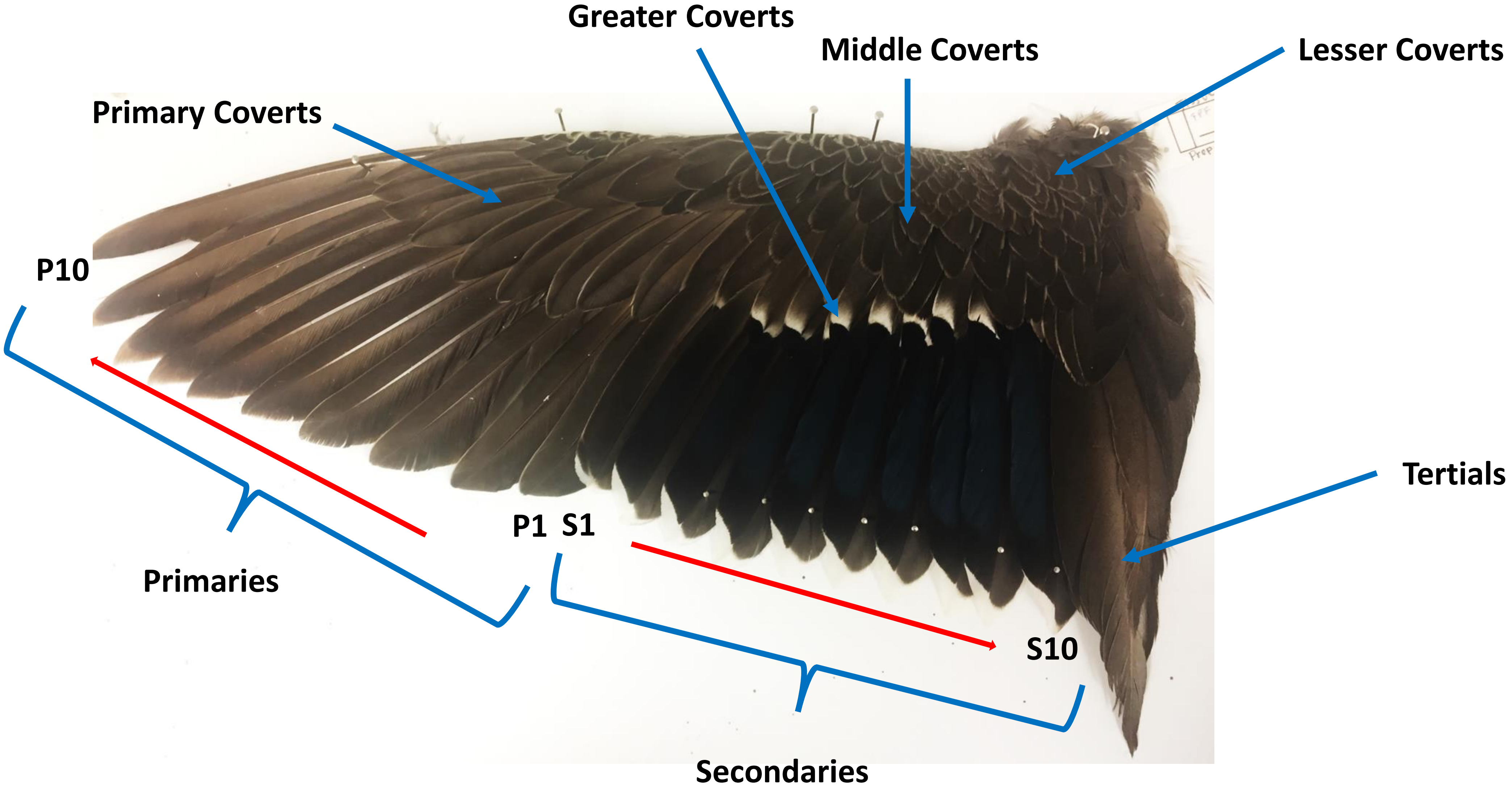

Male Mexican Duck

Mexican and Mallard Plumage Traits Scoring Key

<u>First Year Males</u>

Wing key

MEDU_M

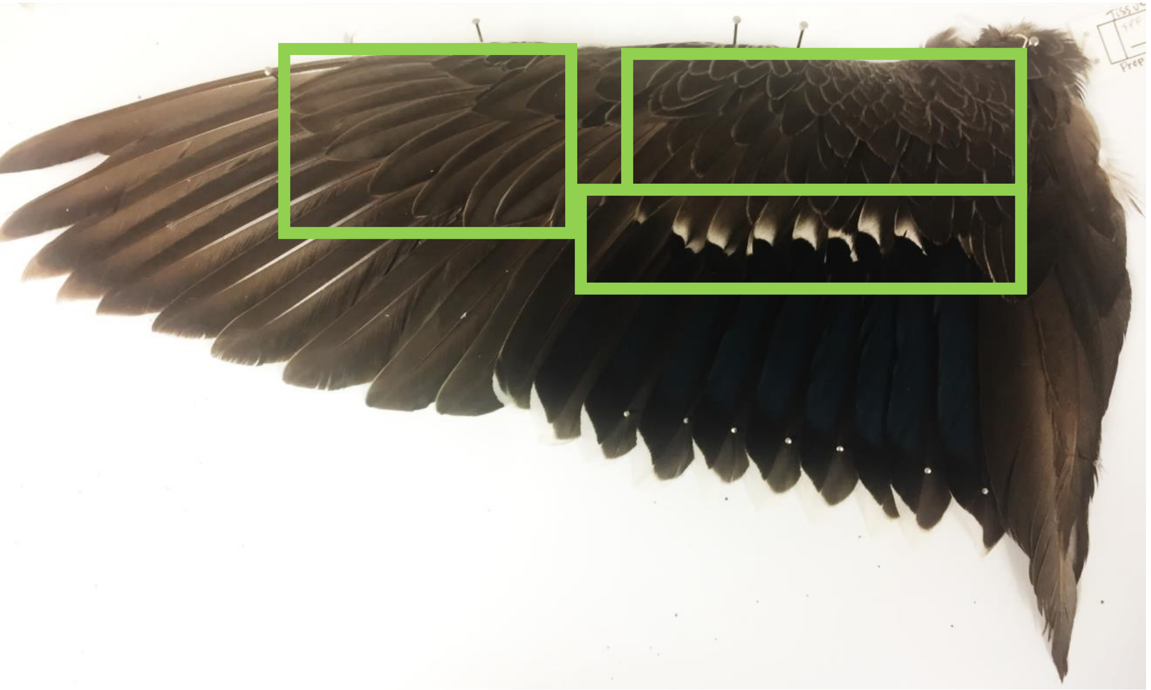

MALL_M

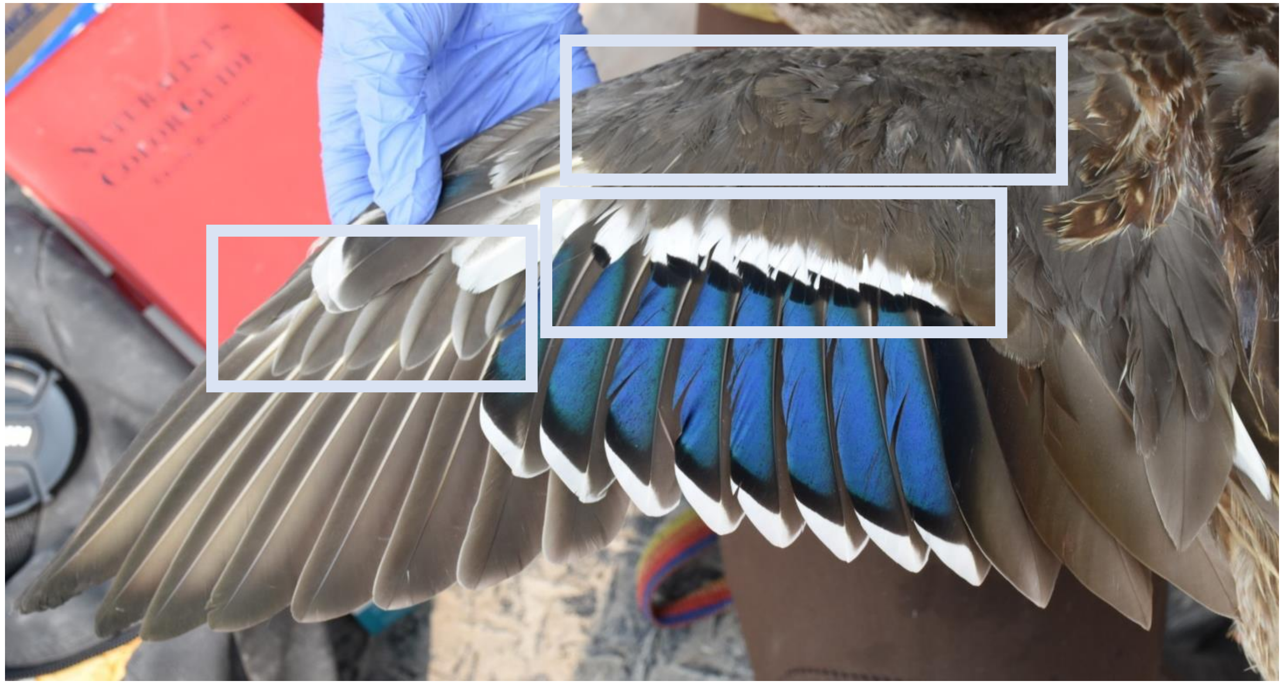

Wing -Subdivision	Trait	Score
Primary covert pattern (edge color)	Buff edged = MEDU	0
	Plain-solid = Hybrid-MALL	1
Lesser covert pattern (edge color)	Buff edged =MEDU	0
	Plain-solid =MALL	1
Greater secondary coverts pattern	Buffy or part white across coverts = MEDU	0
	Complete white across coverts = Hybrid -MALL	1
Speculum color	Green = MEDU	0
	No green = Hybrid -MALL	1

Head key

*Pure Mexican ducks in their northern distribution might present green feathers in their heads

MEDU_M

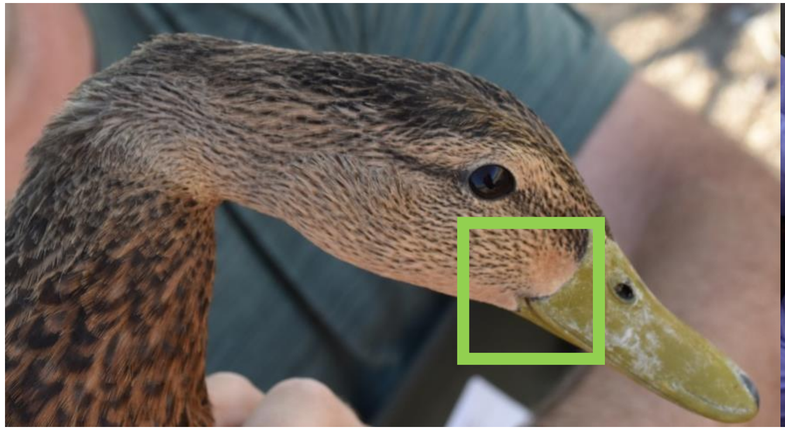

MEDU_M*

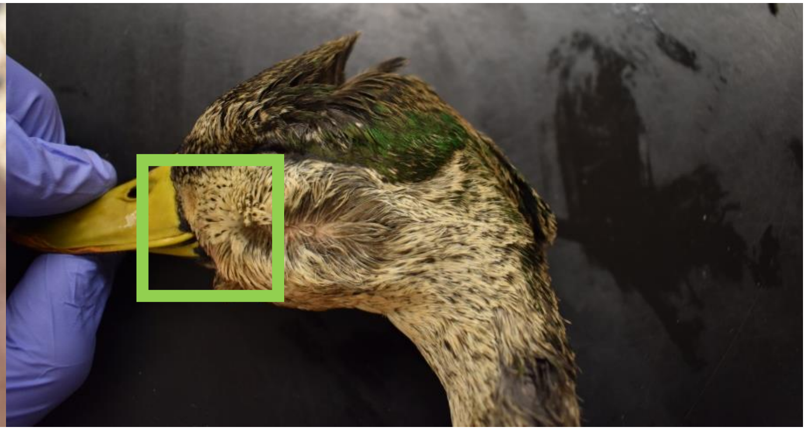

MALL_M

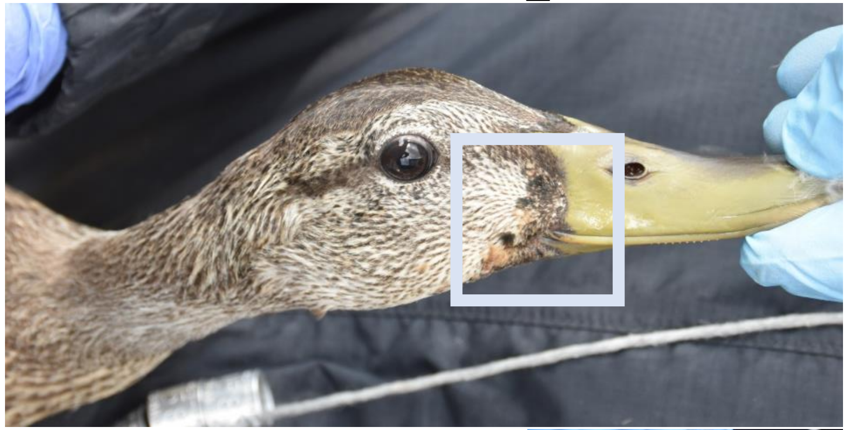

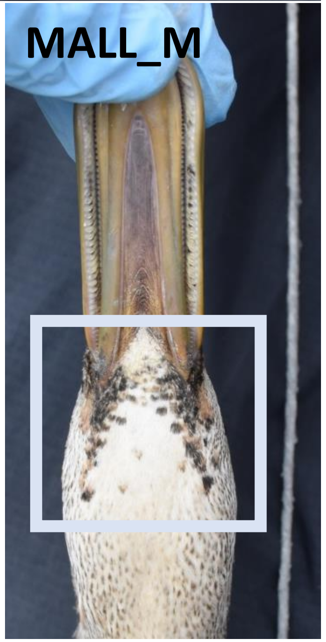

Head-Subdivision	Trait	Score
Percent green in head	No green = MEDU	0
	1-25 %	1
	26-50%	2
	>50% = MALL	3
Overall face and neck *	Slightly patterned = MEDU	0
	Continuously strong patterned = MALL	1
Black spots around bill	Absent = MEDU	0
	Present = MALL	1

Upperparts key

*Pure Mexican ducks in their northern distribution might present green feathers in their heads

MEDU_M

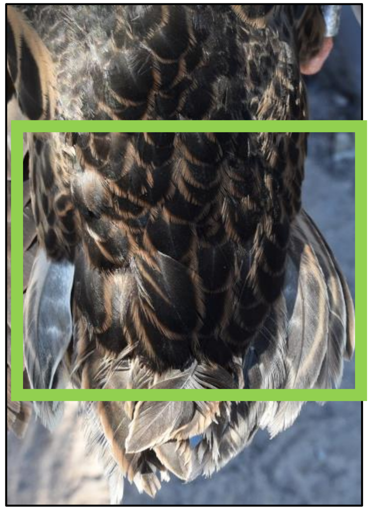

MEDU_M

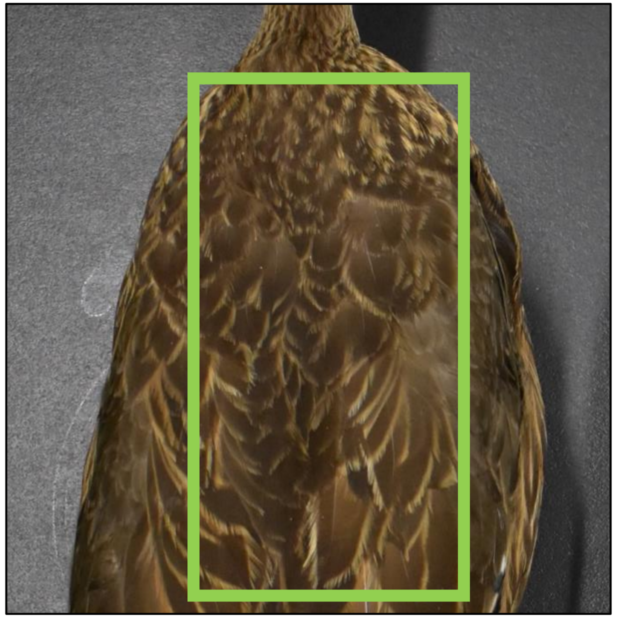

MALL_M

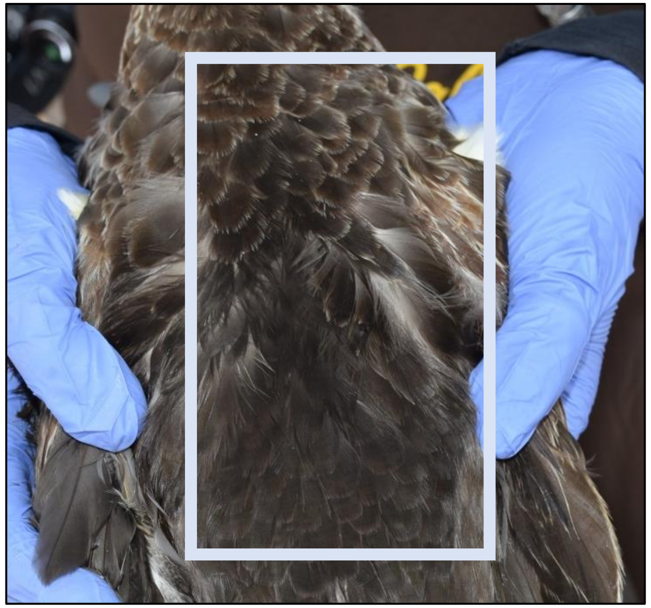

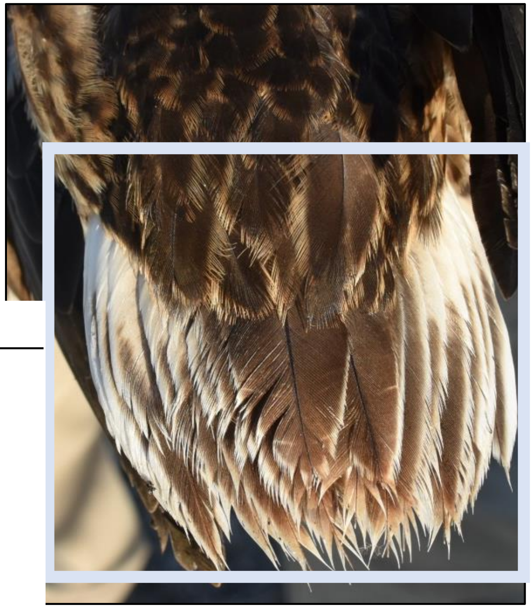

MEDU_M*

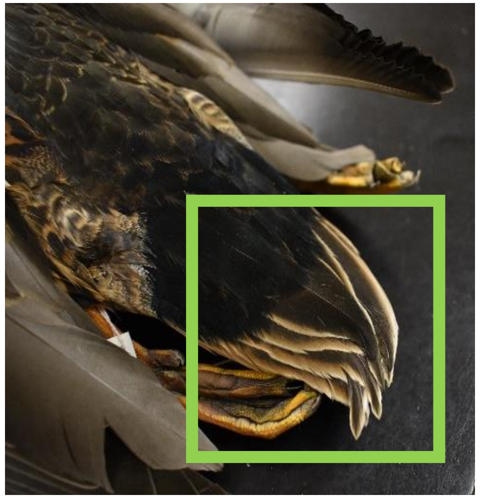

Upperpart- Subdivision	Trait	Score
Overall back feather pattern color*	Chevron patterned or Buff/brown edges = MEDU	0
	Solid or light patterned= MALL	1
Scapular pattern*	Chevron patterned or Buff/brown edges = MEDU	0
	Solid or light patterned = MALL	1
Rump*	Brown w/ buffy chevrons & buffy edges = MEDU	0
	Black w/ rufous chevrons w/ rufous edges = Hybrid	1
	Solid Black = MALL	2
Outer 2 tail feathers (color of outer edges) *	Buff edged = MEDU	0
	White edged = MALL	1
Central tail feathers curl*	Not raised = MEDU	0
	Slightly raised = Hybrid	1
	>Half curl = MALL	2

Underparts Key

*Pure Mexican ducks in their northern distribution might present green feathers in their heads

MEDU_M

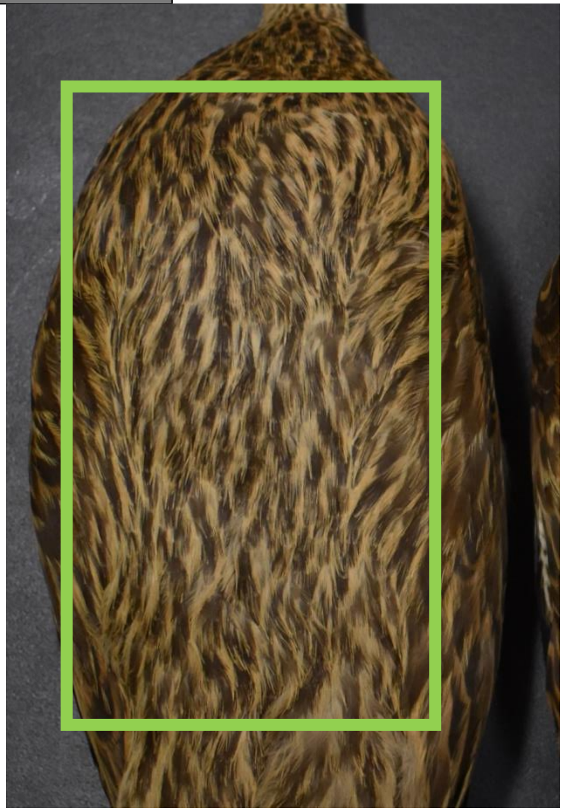

MALL_M

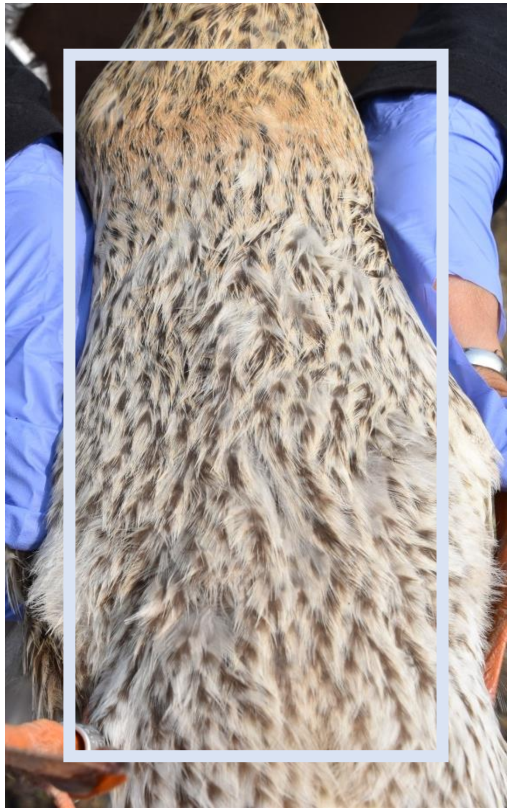

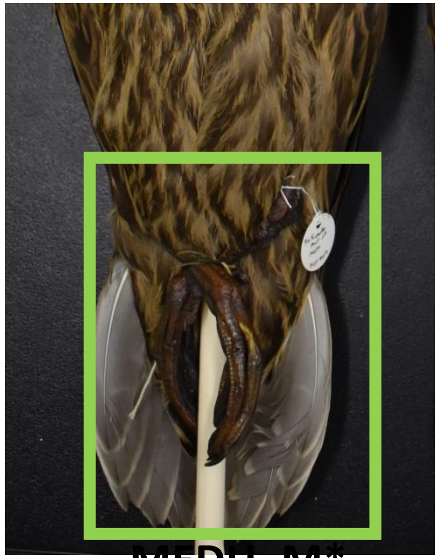

MEDU_M*

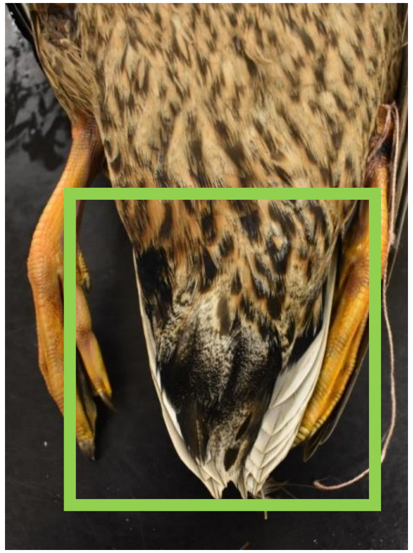

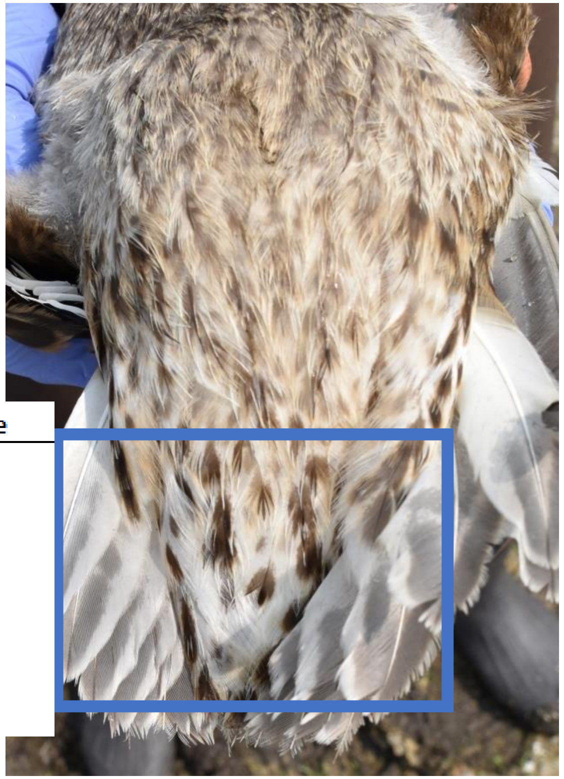

Underparts - Subdivision	Trait	Score
Breast & Belly feather pattern	Strong internal marking = MEDU	0
	Slight internal marking w/whitish edges = MALL	1
Overall breast & belly feather pattern	Uniform = MEDU	0
	Breast & belly different color = MALL	1
Under-tail coverts	Strong. internal marking =MEDU	0
	Spotted patterned to subtle internal marking= MALL	1

Mexican and Mallard Plumage Traits Scoring Key

<u>First Year Females</u>

Wing key

MEDU_F

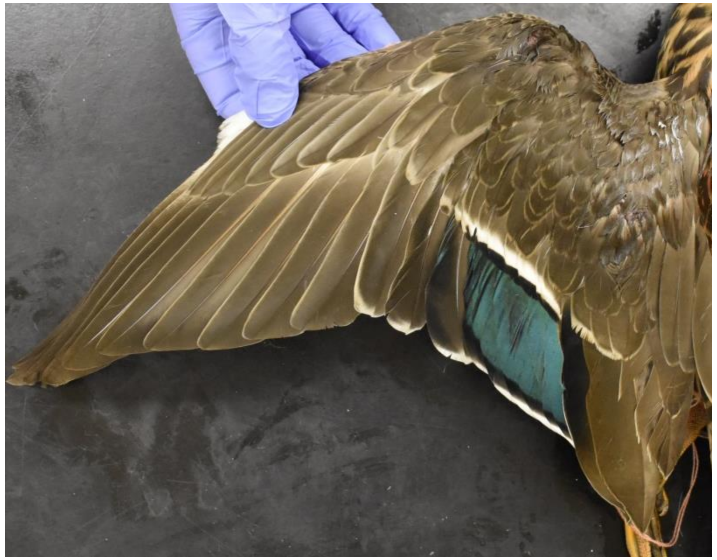

MALL_F

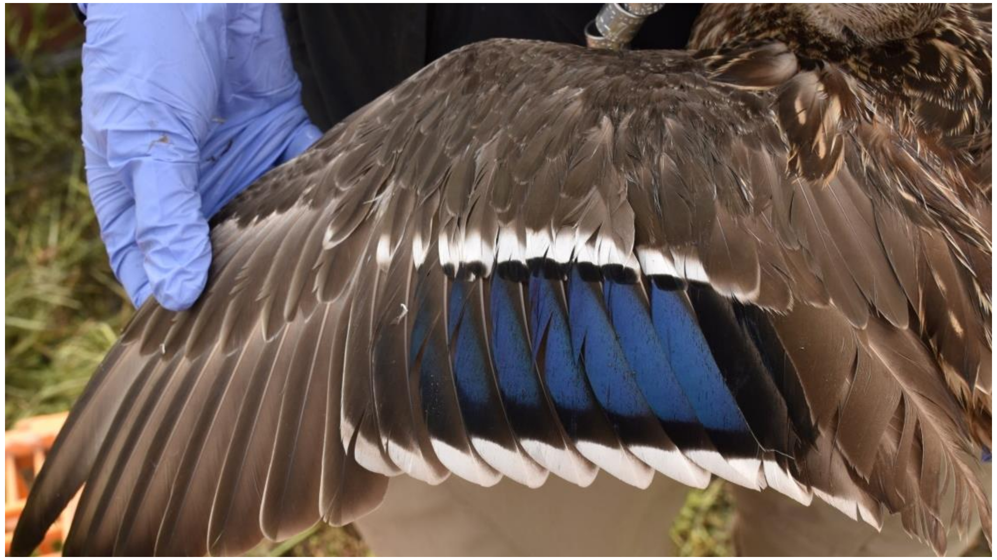

Wing -Subdivision	Trait	Score
Primary covert pattern (edge color)	Buff edged = MEDU	0
	Plain-solid = Hybrid-MALL	1
Lesser covert pattern (edge color)	Buff edged =MEDU	0
	Plain-solid =MALL	1
Greater secondary coverts pattern	Buffy or part white across coverts = MEDU	0
	Complete white across coverts = Hybrid -MALL	1
Speculum color	Green = MEDU	0
	No green = Hybrid -MALL	1

Head key

MEDU_F

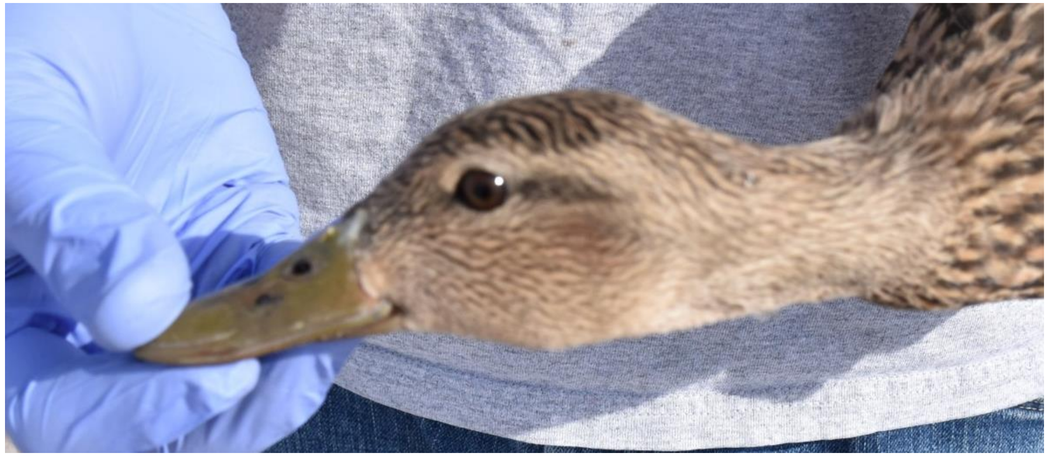

MALL_F

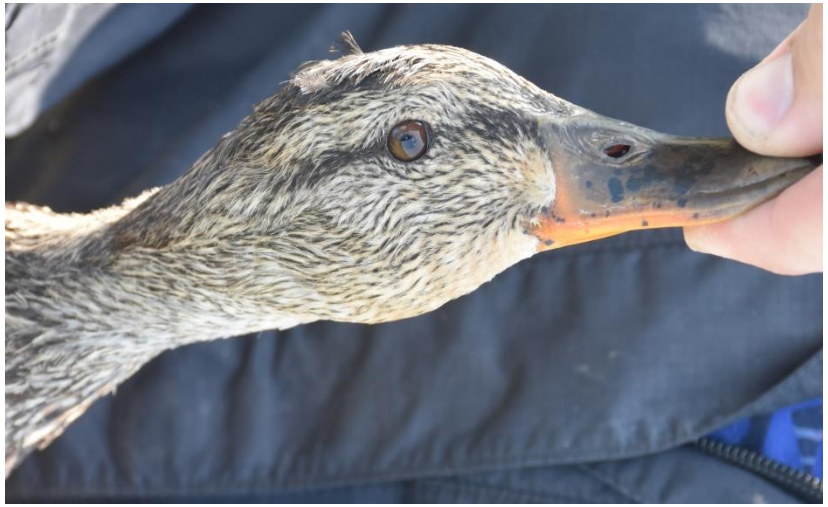

Head-Subdivision	Trait	Score
Overall face and neck *	Slightly patterned = MEDU	0
	Continuously strong patterned = MALL	1

Upperparts key

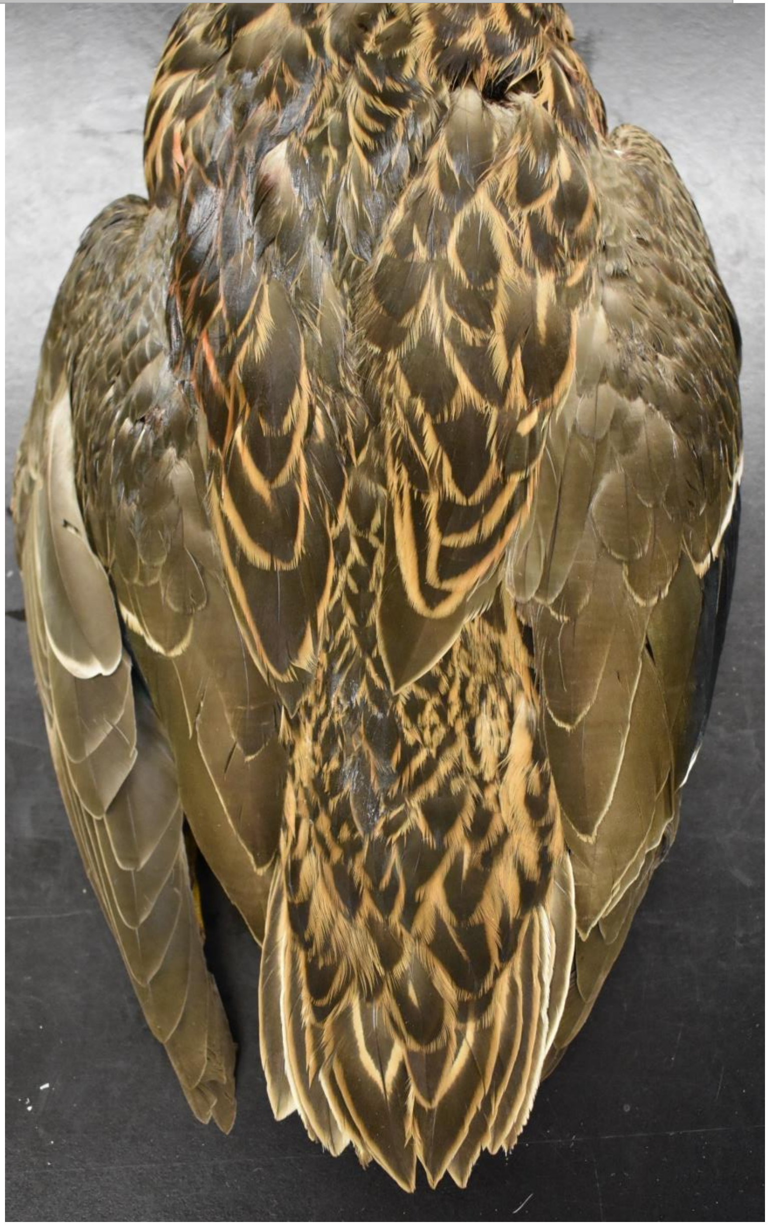

MEDU_F

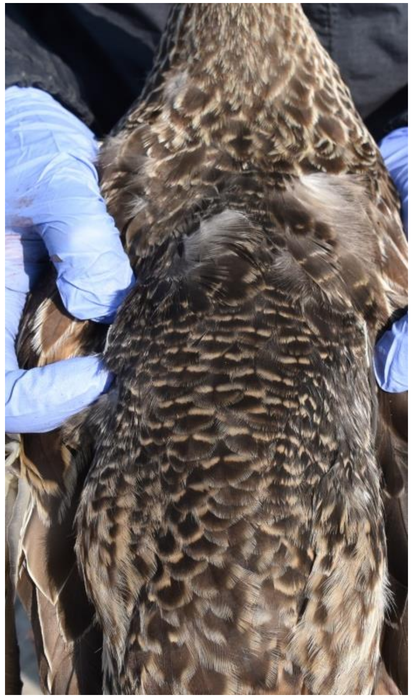

MALL_F

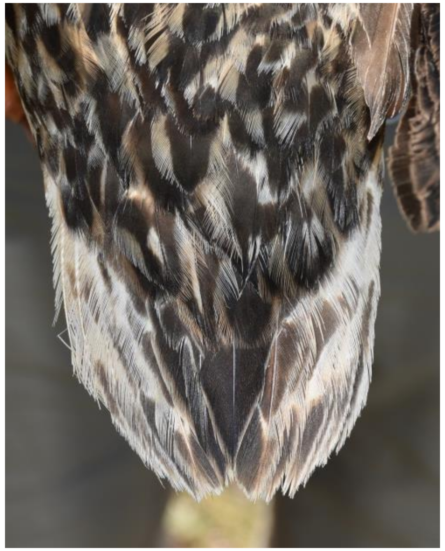

Upperpart- Subdivision	Trait	Score
Overall back feather pattern color*	Chevron patterned or Buff/brown edges = MEDU	0
	Solid or light patterned= MALL	1
Scapular pattern*	Chevron patterned or Buff/brown edges = MEDU	0
	Solid or light patterned = MALL	1
Rump*	Brown w/ buffy chevrons & buffy edges = MEDU	0
	Black w/ rufous chevrons w/ rufous edges = Hybrid	1
Outer 2 tail feathers (color of outer edges) *	Buff edged = MEDU	0
	White edged = MALL	1

Underparts Key

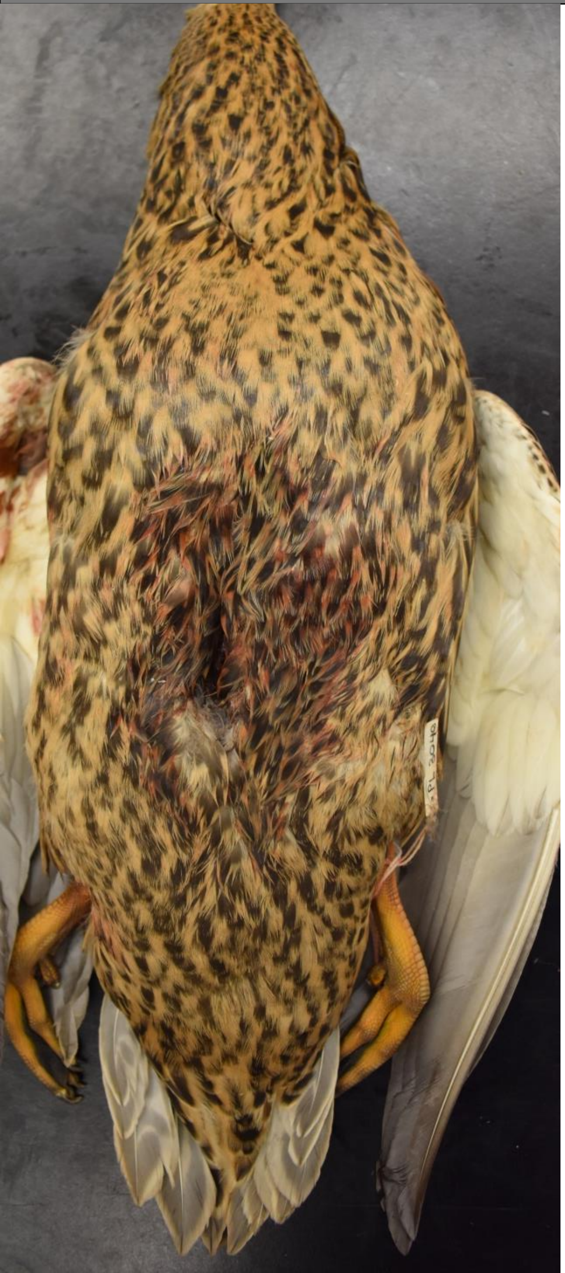

MEDU_F

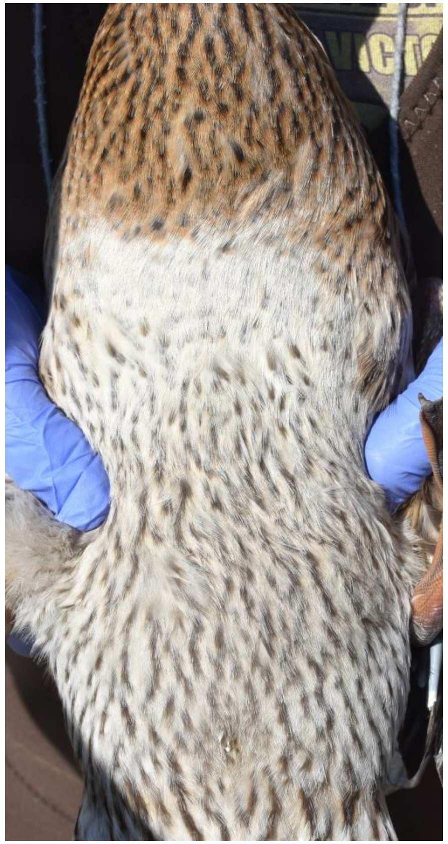

MALL_F

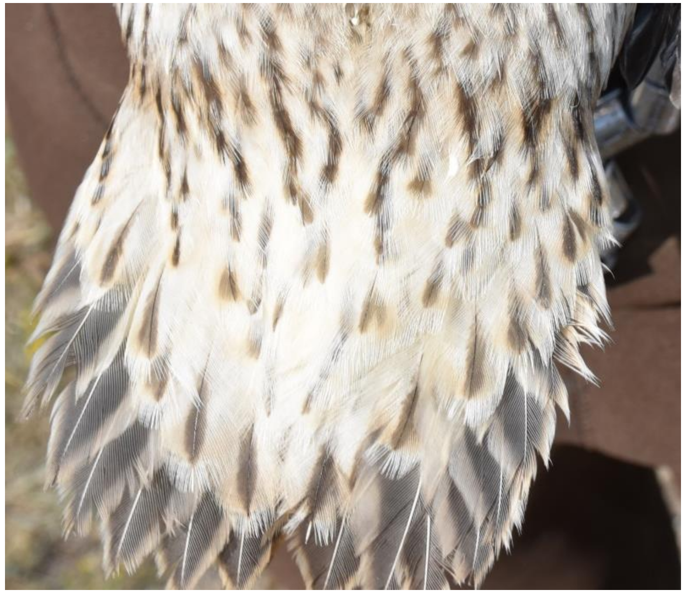

Underparts - Subdivision	Trait	Score
Breast & Belly feather pattern	Strong internal marking = MEDU	0
	Slight internal marking w/whitish edges = MALL	1
Overall breast & belly feather pattern	Uniform = MEDU	0
	Breast & belly different color = MALL	1
Under-tail coverts	Strong. internal marking =MEDU	0
	Spotted patterned to subtle internal marking= MALL	1

Mexican and Mallard Plumage Traits Scoring Key

<u>Adult Males</u>

Wing key

MEDU_M Hybrid_M MALL_M

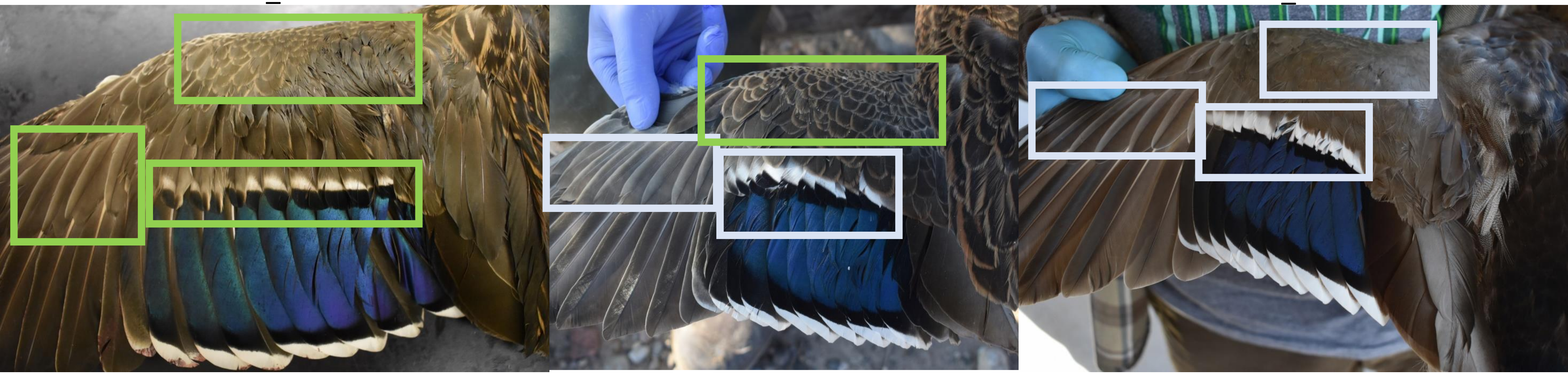

Wing -Subdivision	Trait	Score
Primary covert pattern (edge color)	Buff edged = MEDU	0
	Plain-solid = Hybrid-MALL	1
Lesser covert pattern (edge color)	Buff edged =MEDU	0
	Plain-solid =MALL	1
Greater secondary coverts pattern	Buffy or part white across coverts = MEDU	0
	Complete white across coverts = Hybrid -MALL	1
Speculum color	Green = MEDU	0
	No green = Hybrid -MALL	1

HYBRID -MALL

MEDU

Head key

MEDU_M

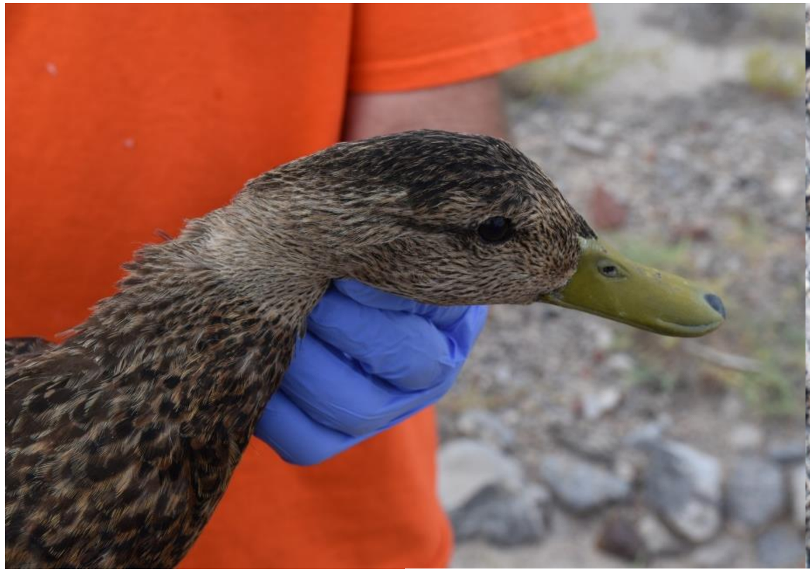

Hybrid_M

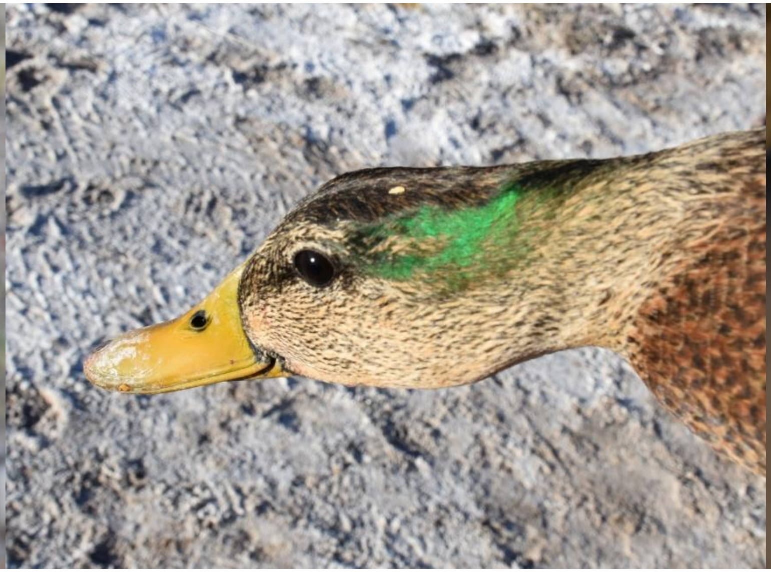

MALL_M

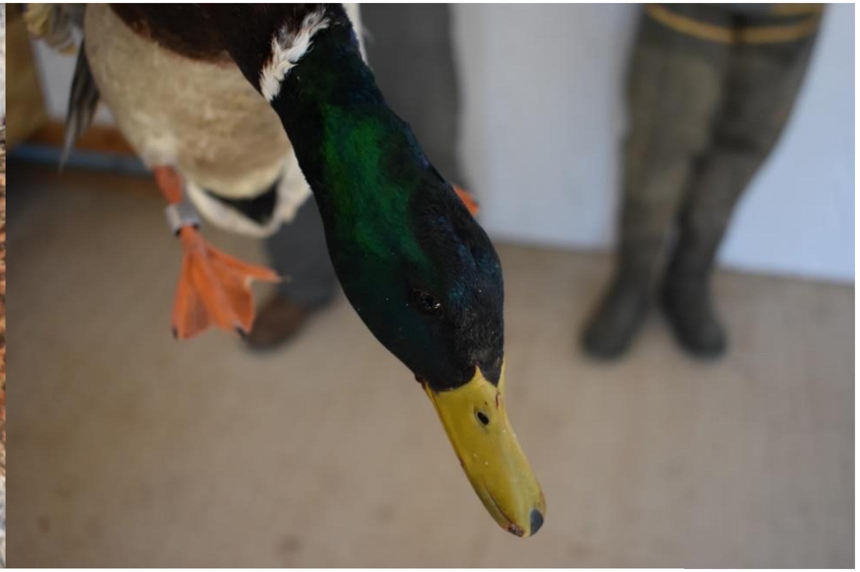

Head-Subdivision	Trait	Score
Percent green in head	No green = MEDU	0
	1-25 %	1
	26-50%	2
	>50% = MALL	3
Overall face and neck *	Slightly patterned = MEDU	0
	Continuously strong patterned = MALL	1
Black spots around bill	Absent = MEDU	0
	Present = MALL	1

Upperparts key

MEDU_M

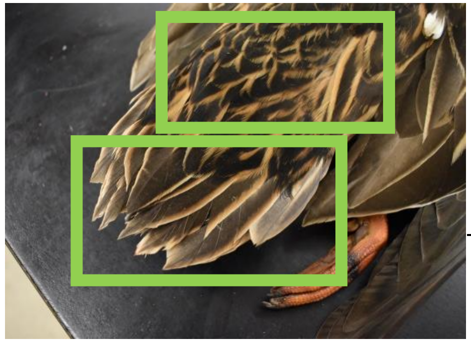

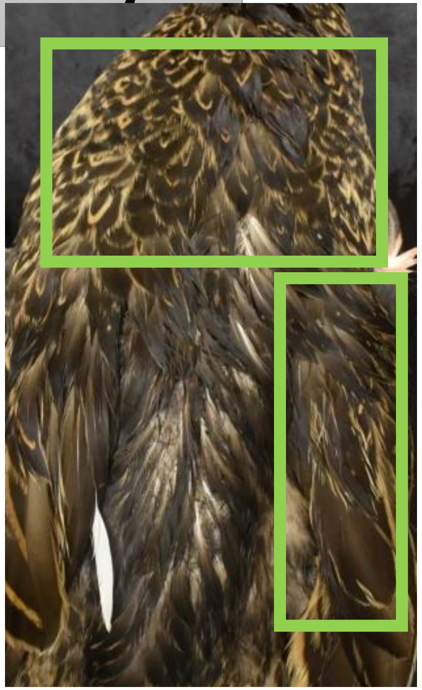

Hybrid_M

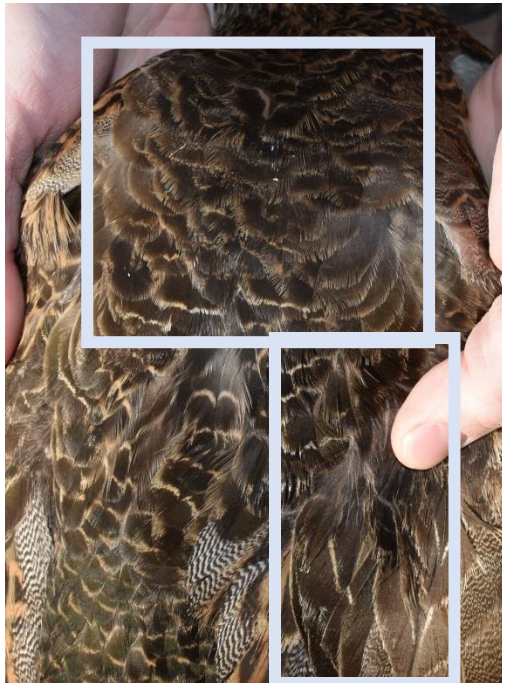

MALL_M

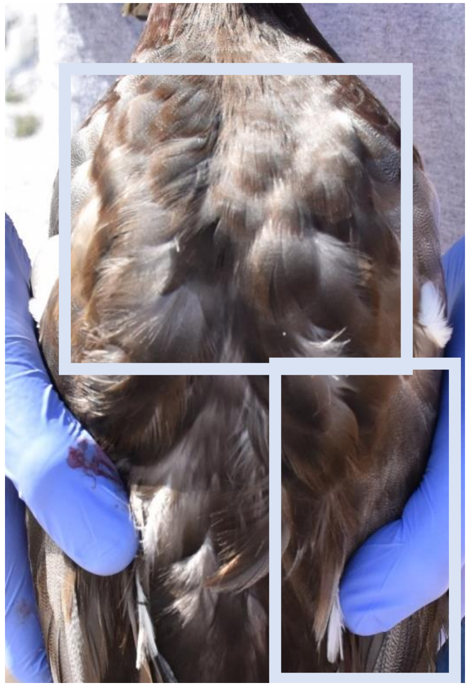

Hybrid_M

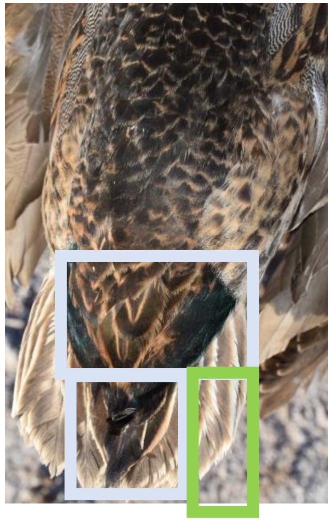

Upperpart- Subdivision	Trait	Score
Overall back feather pattern color*	Chevron patterned or Buff/brown edges = MEDU	0
	Solid or light patterned= MALL	1
Scapular pattern*	Chevron patterned or Buff/brown edges = MEDU	0
	Solid or light patterned = MALL	1
Rump*	Brown w/ buffy chevrons & buffy edges = MEDU	0
	Black w/ rufous chevrons w/ rufous edges = Hybrid	1
	Solid Black = MALL	2
Outer 2 tail feathers (color of outer edges) *	Buff edged = MEDU	0
	White edged = MALL	1
Central tail feathers curl*	Not raised = MEDU	0
	Slightly raised = Hybrid	1
	>Half curl = MALL	2

MALL_M

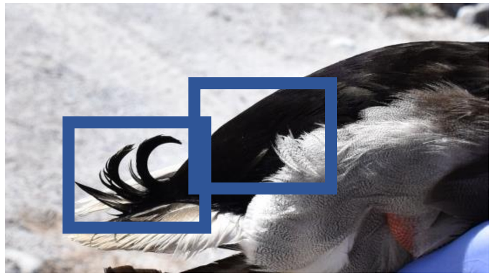

Underparts Key

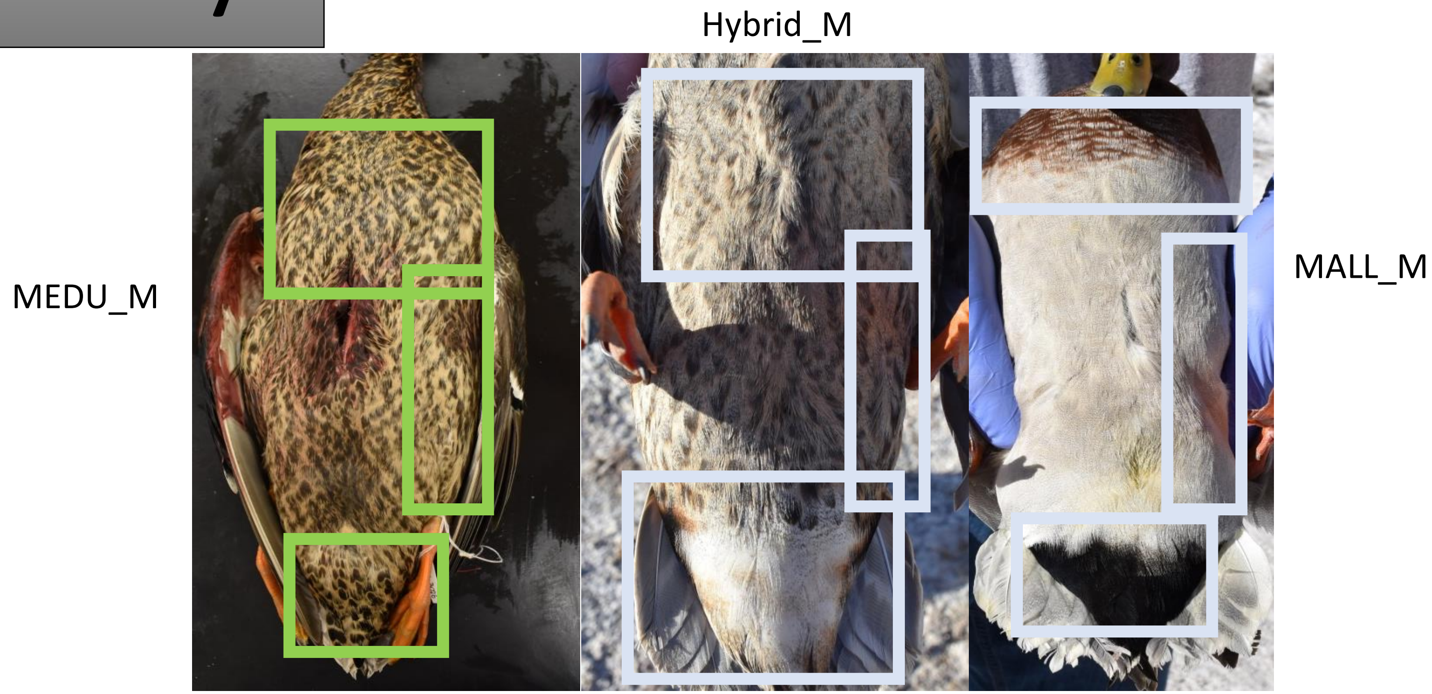

Underparts - Subdivision	Trait	Score
Breast & Belly feather pattern	Strong internal marking = MEDU	0
	Slight internal marking w/whitish edges = MALL	1
Overall breast & belly feather pattern	Uniform = MEDU	0
	Breast & belly different color = MALL	1
Flank feather pattern	Chevron patterned = MEDU	0
	Slight internal marking = MALL	1
Under-tail coverts	Strong. internal marking =MEDU	0
	Spotted patterned to subtle internal marking= MALL	1

Mexican and Mallard Plumage Traits Scoring Key

Adult Females

Wing key

MEDU_F Hybrid_F MALL_F

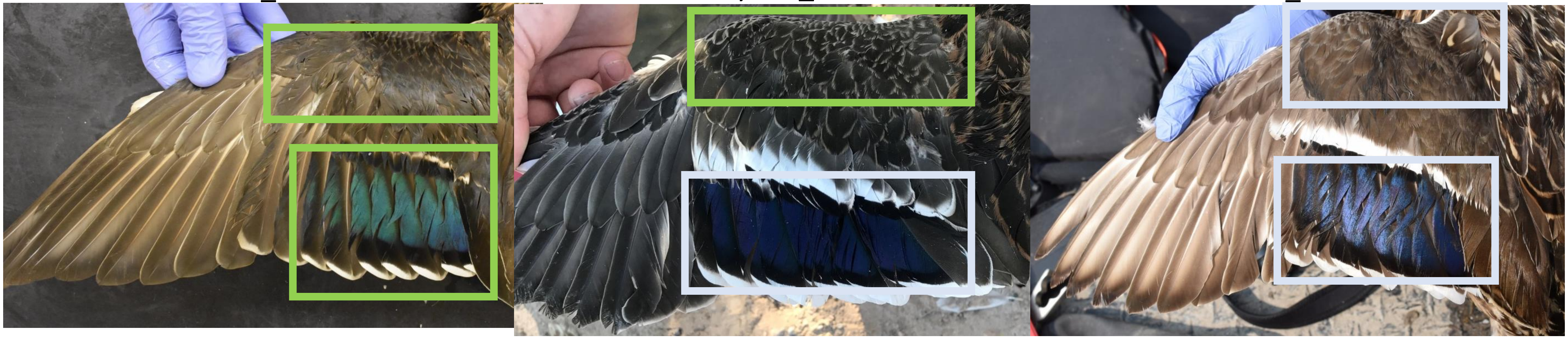

Wing -Subdivision	Trait	Score
Primary covert pattern (edge color)	Buff edged = MEDU	0
	Plain-solid = Hybrid-MALL	1
Lesser covert pattern (edge color)	Buff edged =MEDU	0
	Plain-solid =MALL	1
Greater secondary coverts pattern	Buffy or part white across coverts = MEDU	0
	Complete white across coverts = Hybrid -MALL	1
Speculum color	Green = MEDU	0
	No green = Hybrid -MALL	1

HYBRID -MALL

MEDU

Head key

Hybrid_F MALL_F

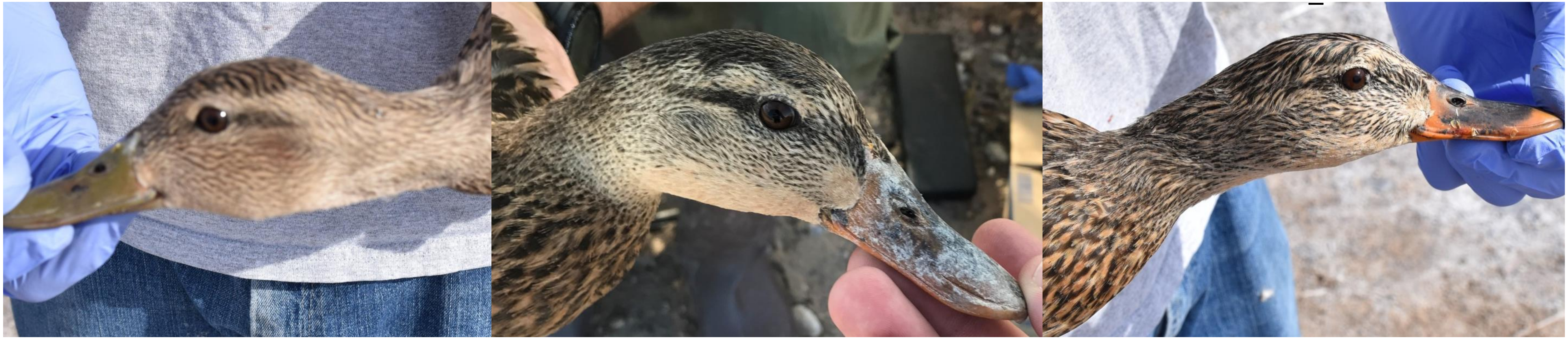

Head-Subdivision	Trait	Score
Overall face and neck *	Slightly patterned = MEDU	0
	Continuously strong patterned = MALL	1

Upperparts key

MEDU_F

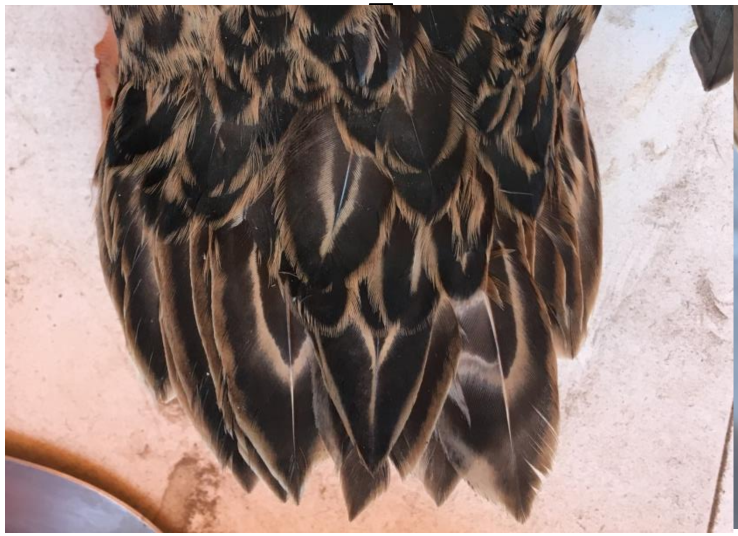

Hybrid_F

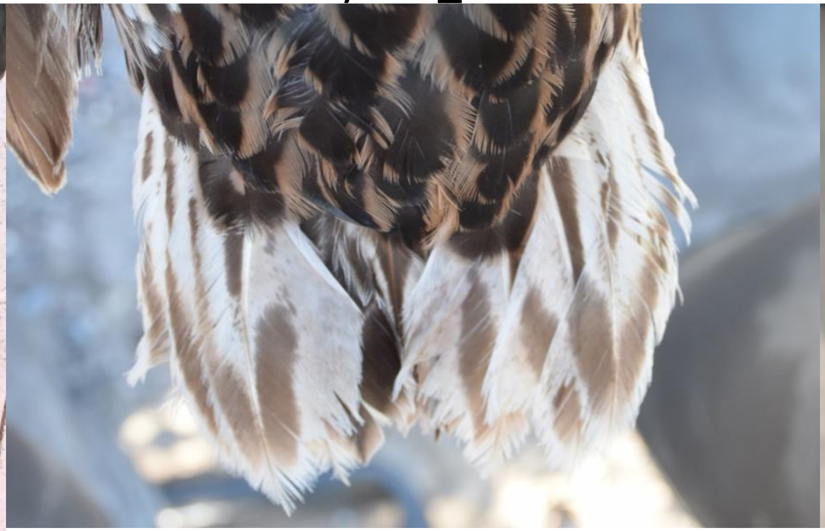

MALL_F

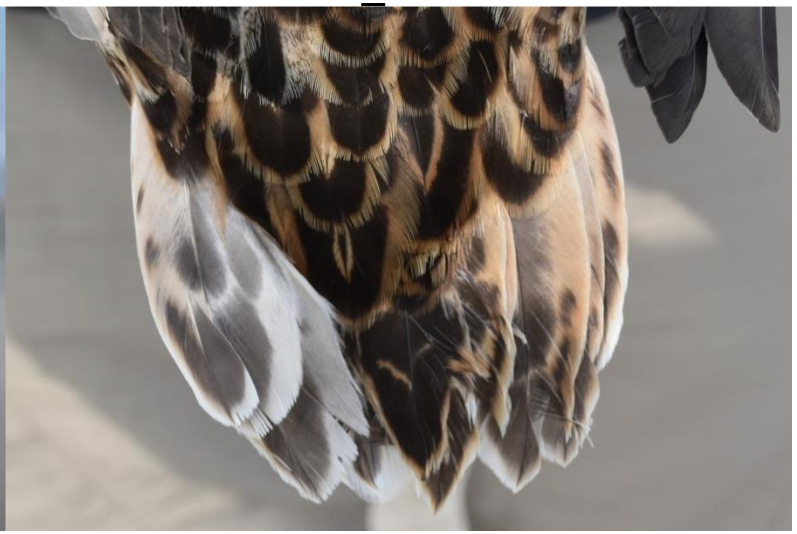

Upperpart- Subdivision	Trait	Score
Outer 2 tail feathers (color of outer edges) *	Buff edged = MEDU	0
	White edged = MALL	1

Underparts Key

MEDU_F | Hybrid_F | MALL_F

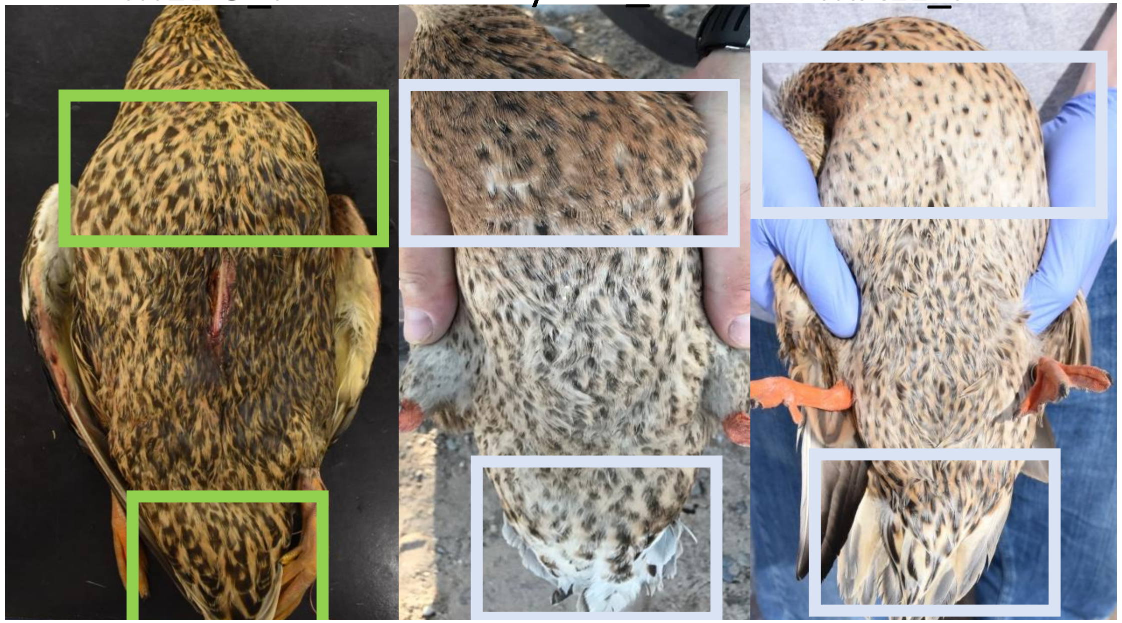

Underparts - Subdivision	Trait	Score
Breast & Belly feather pattern	Strong internal marking = MEDU	0
	Slight internal marking w/whitish edges = MALL	1
Overall breast & belly feather pattern	Uniform = MEDU	0
	Breast & belly different color = MALL	1
Flank feather pattern	Chevron patterned = MEDU	0
	Slight internal marking = MALL	1
Under-tail coverts	Strong. internal marking =MEDU	0
	Spotted patterned to subtle internal marking= MALL	1
