# Supplementary material for: Genomic and morphological data shed light on the complexities of shared ancestry between closely related duck species
Source: Sci Rep. 2022 Jun 17;12:10212. doi: 10.1038/s41598-022-14270-2 (PMC9205961; doi:10.1038/s41598-022-14270-2)
Supplement: Supplementary file 4 — Supplementary Information 4. [file 41598_2022_14270_MOESM4_ESM.docx]

**Title: Genomic and morphological data shed light on the complexities of shared ancestry between closely related duck species**

Joshua I. Brown^1,!^, Flor Hernández^1,!^, Andrew Engilis, Jr.^2,3^, Blanca E. Hernández-Baños^4^, Dan Collins^5^, & Philip Lavretsky^1*^

| **Wing -Subdivision** | **Trait** | **Score** |
| --- | --- | --- |
| Primary covert pattern (edge color) | Buff edged = MEDU | 0 |
|  | Plain-solid = Hybrid-MALL | 1 |
| Lesser covert pattern (edge color) | Buff edged =MEDU | 0 |
|  | Plain-solid =MALL | 1 |
| Greater secondary coverts pattern | Buffy or part white across coverts = MEDU | 0 |
|  | Complete white across coverts = Hybrid -MALL | 1 |
| Speculum color | Green = MEDU | 0 |
|  | No green = Hybrid -MALL | 1 |

**Mexican and Mallard Plumage Traits Scoring Key – First Year Females**

**Band #: _________________ Date: _____________ Locality:_________ Recorder:___________**

| **Head-Subdivision** | | **Trait** | | **Score** | |
| --- | --- | --- | --- | --- | --- |
| Overall face and neck * | Slightly patterned = MEDU | | | 0 | |
|  | Continuously strong patterned = MALL | | | 1 | |
|  |  | | | |  |
| **Upperpart- Subdivision** | **Trait** | | | | **Score** |
| Overall back feather pattern color* | Chevron patterned or Buff/brown edges = MEDU | | 0 | | |
|  | Solid or light patterned= MALL | | 1 | | |
| Scapular pattern* | Chevron patterned or Buff/brown edges = MEDU | | 0 | | |
|  | Solid or light patterned = MALL | | 1 | | |
| Rump* | Brown w/ buffy chevrons & buffy edges = MEDU | | 0 | | |
|  | Black w/ rufous chevrons w/ rufous edges = Hybrid | | 1 | | |
|  | Solid Black = MALL | | 2 | | |
| Outer 2 tail feathers (color of outer | Buff edged = MEDU | | 0 | | |
| edges) * | White edged = MALL | | 1 | | |

| **Underparts - Subdivision** | **Trait** | **Score** |
| --- | --- | --- |
| Breast & Belly feather pattern | Strong internal marking = MEDU | 0 |
|  | Slight internal marking w/whitish edges = MALL | 1 |
| Overall breast & belly feather | Uniform = MEDU | 0 |
| pattern | Breast & belly different color = MALL | 1 |
| Under-tail coverts | Strong. internal marking =MEDU | 0 |
|  | Spotted patterned to subtle internal marking= MALL | 1 |

|  | \| PS score ≤ 4 = MEDU \| \| --- \| \| PS score ≥ 5 = MALL/HYB \|   **Total Sum of Phenotypic Scores (PS):** | ____ |
| --- | --- | --- | --- | --- |

| **Conclusion:** | ___________ |
| --- | --- |

**Mexican and Mallard Plumage Traits Scoring Key – First Year Males**

**Band #: _________________ Date: _____________ Locality: ___________ Recorder:___________**

| **Wing -Subdivision** | **Trait** | **Score** |
| --- | --- | --- |
| Primary covert pattern (edge color) | Buff edged = MEDU | 0 |
|  | Plain-solid = Hybrid-MALL | 1 |
| Lesser covert pattern (edge color) | Buff edged =MEDU | 0 |
|  | Plain-solid =MALL | 1 |
| Greater secondary coverts pattern | Buffy or part white across coverts = MEDU | 0 |
|  | Complete white across coverts = Hybrid -MALL | 1 |
| Speculum color | Green = MEDU | 0 |
|  | No green = Hybrid -MALL | 1 |

| **Head-Subdivision** | | **Trait** | | **Score** | |
| --- | --- | --- | --- | --- | --- |
| Percent green in head | No green = MEDU | | | 0 | |
|  | 1-25 % | | | 1 | |
|  | 26-50% | | | 2 | |
|  | >50% = MALL | | | 3 | |
| Overall face and neck * | Slightly patterned = MEDU | | | 0 | |
|  | Continuously strong patterned = MALL | | | 1 | |
| Black spots around bill | Absent = MEDU | | | | 0 |
|  | Present = MALL | | | | 1 |
| **Upperpart- Subdivision** | **Trait** | | | | **Score** |
| Overall back feather pattern color* | Chevron patterned or Buff/brown edges = MEDU | | 0 | | |
|  | Solid or light patterned= MALL | | 1 | | |
| Scapular pattern* | Chevron patterned or Buff/brown edges = MEDU | | 0 | | |
|  | Solid or light patterned = MALL | | 1 | | |
| Rump* | Brown w/ buffy chevrons & buffy edges = MEDU | | 0 | | |
|  | Black w/ rufous chevrons w/ rufous edges = Hybrid | | 1 | | |
|  | Solid Black = MALL | | 2 | | |
| Outer 2 tail feathers (color of outer | Buff edged = MEDU | | 0 | | |
| edges) * | White edged = MALL | | 1 | | |
| Central tail feathers curl* | Not raised = MEDU | | 0 | | |
|  | Slightly raised = Hybrid | | 1 | | |
|  | >Half curl = MALL | | 2 | | |

| **Underparts - Subdivision** | **Trait** | **Score** |
| --- | --- | --- |
| Breast & Belly feather pattern | Strong internal marking = MEDU | 0 |
|  | Slight internal marking w/whitish edges = MALL | 1 |
| Overall breast & belly feather | Uniform = MEDU | 0 |
| pattern | Breast & belly different color = MALL | 1 |
| Flank feather pattern | Chevron patterned = MEDU | 0 |
|  | Slight internal marking = MALL | 1 |
| Under-tail coverts | Strong. internal marking =MEDU | 0 |
|  | Spotted patterned to subtle internal marking= MALL | 1 |
|  |  |  |
|  |  |  |
|  |  |  |
|  |  |  |
|  |  |  |
|  |  |  |
|  |  |  |

|  | \| PS score ≤ 4 = MEDU \| \| --- \| \| PS score ≥ 5 = MALL/HYB \|   **Total Sum of Phenotypic Scores (PS):** | ____ |
| --- | --- | --- | --- | --- |

| **Conclusion:** | ___________ |
| --- | --- |

| **Wing -Subdivision** | **Trait** | **Score** |
| --- | --- | --- |
| Primary covert pattern (edge color) | Buff edged = MEDU | 0 |
|  | Plain-solid = Hybrid-MALL | 1 |
| Lesser covert pattern (edge color) | Buff edged =MEDU | 0 |
|  | Plain-solid =MALL | 1 |
| Greater secondary coverts pattern | Buffy or part white across coverts = MEDU | 0 |
|  | Complete white across coverts = Hybrid -MALL | 1 |
| Speculum color | Green = MEDU | 0 |
|  | No green = Hybrid -MALL | 1 |

**Mexican and Mallard Plumage Traits Scoring Key – Adult Females**

**Band #: _________________ Date: _____________ Locality:_________ Recorder:___________**

| **Head-Subdivision** | | **Trait** | | **Score** | |
| --- | --- | --- | --- | --- | --- |
| Overall face and neck * | Slightly patterned = MEDU | | | 0 | |
|  | Continuously strong patterned = MALL | | | 1 | |
|  |  | | | |  |
| **Upperpart- Subdivision** | **Trait** | | | | **Score** |
| Outer 2 tail feathers (color of outer | Buff edged = MEDU | | 0 | | |
| edges) * | White edged = MALL | | 1 | | |

| **Underparts - Subdivision** | **Trait** | **Score** |
| --- | --- | --- |
| Breast & Belly feather pattern | Strong internal marking = MEDU | 0 |
|  | Slight internal marking w/whitish edges = MALL | 1 |
| Overall breast & belly feather | Uniform = MEDU | 0 |
| pattern | Breast & belly different color = MALL | 1 |
| Flank feather pattern | Chevron patterned = MEDU | 0 |
|  | Slight internal marking = MALL | 1 |
| Under-tail coverts | Strong. internal marking =MEDU | 0 |
|  | Spotted patterned to subtle internal marking= MALL | 1 |

|  | \| PS score ≤ 4 = MEDU \| \| --- \| \| PS score ≥ 5 = MALL/HYB \|   **Total Sum of Phenotypic Scores (PS):** | ____ |
| --- | --- | --- | --- | --- |

| **Conclusion:** | ___________ |
| --- | --- |

**Mexican and Mallard Plumage Traits Scoring Key – Adult Males**

**Band #: _________________ Date: _____________ Locality: ___________ Recorder:___________**

| **Wing -Subdivision** | **Trait** | **Score** |
| --- | --- | --- |
| Primary covert pattern (edge color) | Buff edged = MEDU | 0 |
|  | Plain-solid = Hybrid-MALL | 1 |
| Lesser covert pattern (edge color) | Buff edged =MEDU | 0 |
|  | Plain-solid =MALL | 1 |
| Greater secondary coverts pattern | Buffy or part white across coverts = MEDU | 0 |
|  | Complete white across coverts = Hybrid -MALL | 1 |
| Speculum color | Green = MEDU | 0 |
|  | No green = Hybrid -MALL | 1 |

| **Head-Subdivision** | | **Trait** | | **Score** | |
| --- | --- | --- | --- | --- | --- |
| Percent green in head | No green = MEDU | | | 0 | |
|  | 1-25 % | | | 1 | |
|  | 26-50% | | | 2 | |
|  | >50% = MALL | | | 3 | |
| Overall face and neck * | Slightly patterned = MEDU | | | 0 | |
|  | Continuously strong patterned = MALL | | | 1 | |
| Black spots around bill | Absent = MEDU | | | | 0 |
|  | Present = MALL | | | | 1 |
| **Upperpart- Subdivision** | **Trait** | | | | **Score** |
| Overall back feather pattern color* | Chevron patterned or Buff/brown edges = MEDU | | 0 | | |
|  | Solid or light patterned= MALL | | 1 | | |
| Scapular pattern* | Chevron patterned or Buff/brown edges = MEDU | | 0 | | |
|  | Solid or light patterned = MALL | | 1 | | |
| Rump* | Brown w/ buffy chevrons & buffy edges = MEDU | | 0 | | |
|  | Black w/ rufous chevrons w/ rufous edges = Hybrid | | 1 | | |
|  | Solid Black = MALL | | 2 | | |
| Outer 2 tail feathers (color of outer | Buff edged = MEDU | | 0 | | |
| edges) * | White edged = MALL | | 1 | | |
| Central tail feathers curl* | Not raised = MEDU | | 0 | | |
|  | Slightly raised = Hybrid | | 1 | | |
|  | >Half curl = MALL | | 2 | | |

| **Underparts - Subdivision** | **Trait** | **Score** |
| --- | --- | --- |
| Breast & Belly feather pattern | Strong internal marking = MEDU | 0 |
|  | Slight internal marking w/whitish edges = MALL | 1 |
| Overall breast & belly feather | Uniform = MEDU | 0 |
| pattern | Breast & belly different color = MALL | 1 |
| Flank feather pattern | Chevron patterned = MEDU | 0 |
|  | Slight internal marking = MALL | 1 |
| Under-tail coverts | Strong. internal marking =MEDU | 0 |
|  | Spotted patterned to subtle internal marking= MALL | 1 |

|  |
| --- |

|  | \| PS score ≤ 4 = MEDU \| \| --- \| \| PS score ≥ 5 = MALL/HYB \|   **Total Sum of Phenotypic Scores (PS):** | ____ |
| --- | --- | --- | --- | --- |

| **Conclusion:** | ___________ |
| --- | --- |
